# Epistasis and pleiotropy constrain the evolution of cross-reactive breadth in a broadly neutralizing influenza antibody

**DOI:** 10.1371/journal.pbio.3004021

**Published:** 2026-09-28

**Authors:** Katrine E. Dailey, Yiquan Wang, Qi Wen Teo, Chaoyang Wang, Huibin Lv, Ruipeng Lei, Meixuan Tong, Lucia A. Rodriguez, Letianchu Wang, Nicholas C. Wu

**Affiliations:** 1 Department of Biochemistry, University of Illinois Urbana-Champaign, Urbana, Illinois, United States of America; 2 Department of Infectious Diseases and Immunology, University of Florida, Gainesville, Florida, United States of America; 3 Carl R. Woese Institute for Genomic Biology, University of Illinois Urbana-Champaign, Urbana, Illinois, United States of America; 4 Center for Biophysics and Quantitative Biology, University of Illinois Urbana-Champaign, Urbana, Illinois, United States of America; 5 Carle Illinois College of Medicine, University of Illinois Urbana-Champaign, Urbana, Illinois, United States of America; Fred Hutchinson Cancer Research Center, UNITED STATES OF AMERICA

## Abstract

The highly conserved influenza virus hemagglutinin (HA) stem domain is a major target for broadly neutralizing antibodies (bnAbs). However, despite being discovered more than a decade ago, the IGHV1-69-encoded CR9114 remains the only HA stem bnAb that cross-reacts with both influenza A and B viruses. To investigate the constraints on the breadth evolution of CR9114, this study performs four deep mutational scanning experiments to compare the binding affinity landscapes of the germline and somatic CR9114 against H1 HA, H3 HA, and influenza B virus HA. Many mutations that minimally affect or even improve the H1 HA binding are detrimental for binding to H3 HA and influenza B virus HA. We further reveal the prevalence of epistasis in IGHV1-69 HA stem bnAbs. Overall, our findings provide a mechanistic explanation for the scarcity of HA stem bnAbs with cross-reactivity against both influenza A and B viruses, and have important implications for developing broadly protective influenza vaccines.

## Abstract 2

Broadly neutralizing flu antibodies able to recognize both influenza A and B viruses are exceptionally rare. This study shows that mutations which preserve or improve binding to one flu virus can weaken recognition of others, constraining antibody cross-reactive breadth.

## Introduction

Influenza viruses are classified into four types (A–D), with types A and B posing major global public health concerns. Hemagglutinin (HA), which consists of a hypervariable head domain atop a conserved stem domain [1], is the major surface antigen of influenza A and B viruses. Influenza A virus HA is categorized into 19 subtypes (H1–H19) that fall into two groups. Group 1 includes H1, H2, H5, H6, H8, H9, H11, H12, H13, H16, H17, H18 and H19, whereas group 2 includes H3, H4, H7, H10, H14, and H15 [2]. Similarly, influenza B virus HA (BHA) has two lineages, B/Yamagata/16/1988-like and B/Victoria/2/1987-like, although the former has not been detected since the coronavirus disease 2019 (COVID-19) pandemic [3]. Each year, H1 and H3 subtypes of influenza A virus, along with influenza B virus, cause seasonal influenza epidemics, leading to widespread illness, absenteeism, and loss of life [4]. Additionally, influenza A virus has caused five pandemics in the past 150 years with a high mortality burden, and remains at high risk for causing a future pandemic [5–8].

Seasonal influenza vaccines, which are designed to elicit neutralizing HA antibodies, are currently the most effective preventive measures against influenza viruses. However, despite annual updates, the rapid antigenic drift of HA in seasonal influenza viruses sometimes leads to antigenic mismatch between the vaccine and circulating strains, resulting in low vaccine effectiveness in some years [9]. Furthermore, seasonal influenza vaccines do not confer protection against zoonotic subtypes that are antigenically distinct from human strains [10]. The limited heterosubtypic protection of seasonal influenza vaccines is largely attributable to the antibody responses being mostly directed against the hypervariable head domain, which is immunodominant over the conserved stem domain [11]. As a result, a major goal of the influenza research field is to develop more broadly protective vaccines.

HA stem has been the major target for the development of broadly protective influenza vaccines, owing to the discovery of broadly neutralizing antibodies (bnAbs) to the HA stem almost two decades ago [12–14]. Among hundreds of HA stem bnAbs that have been identified and characterized, CR9114 has the largest cross-reactivity breadth [15]. CR9114 not only binds to both group 1 and 2 HAs, but also BHA [15]. By contrast, none of the other known HA stem bnAbs have demonstrated the ability to cross-react with both influenza A and B virus HAs. The rarity of HA stem bnAbs exhibiting cross-reactivity against influenza A and B virus HAs may be attributed to the as-yet-unknown constraints of the evolutionary pathways required for their breadth evolution. By focusing on 16 somatic hypermutations in CR9114, a previous study has shown that the inferred germline of CR9114 (hereinafter referred to as germline CR9114) is specific to H1 HA and subsequently evolved breadth by acquiring affinity to H3 HA via somatic hypermutations, then to BHA [16]. However, it is unclear how the ability of CR9114 to evolve such exceptional breadth would be impacted by an alternative evolutionary pathway involving other potential somatic hypermutations.

In this study, we performed a series of Tite-Seq [17] experiments to quantify the effects of almost all possible single amino acid mutations in the heavy chain variable domain (V_H_) of the germline and somatic CR9114 against different HAs. We found that the mutational tolerance of somatic CR9114 was the highest against H1 HA and lowest against BHA. Consequently, a number of mutations neutral or beneficial to germline or somatic CR9114 against H1 HA could abolish binding to H3 HA and BHA. These findings suggested that the evolution of broad reactivity by CR9114 is highly constrained and easily derailed due to epistasis and pleiotropy. Throughout this study, antibody residues are numbered using Kabat nomenclature.

## Results

### Deep mutational scanning of the germline and somatic CR9114

To probe the evolutionary landscape of CR9114, we aimed to determine the binding affinity of all possible single amino acid mutations of the germline and somatic CR9114 against different HAs (Fig 1). Briefly, site-saturation mutagenesis was performed on the heavy chain variable domain of the germline and somatic CR9114. We did not include any mutations in the light chain, since it is not involved in binding [15]. Next-generation sequencing indicated that the frequencies of the mutations were fairly even (S1 Fig). The mutant libraries were then displayed on the yeast surface and selected for binding against different HAs. Since germline CR9114 does not cross-react with group 2 HAs and BHA [16], the mutant library of the germline CR9114 was selected against a recombinant HA stem construct that was designed based on H1N1 A/Brisbane/59/2007 HA (hereinafter referred to as H1 stem) [18]. On the other hand, the mutant library of somatic CR9114 was selected against H1 stem, H3N2 A/Singapore/INFIMH-16-0019/2016 (H3/Sing16) HA, and influenza B/Lee/1940 (B/Lee40) HA. The apparent binding affinity (*K*_*D,app*_) of each mutation was then quantified by Tite-Seq [17], which combined fluorescence-activated cell sorting (FACS) and next-generation sequencing (Fig 2 and S1 Data). For each mutation, we used −Δlog_10_ *K*_*D,app*_, to quantify its effect on binding affinity. The −Δlog_10_ *K*_*D,app*_ value represented the difference between the Δlog_10_ *K*_*D,app*_ of a given mutation and that of the corresponding wild type (i.e., germline or somatic CR9114). A lower −Δlog_10_ *K*_*D,app*_ value indicated a more detrimental effect on binding affinity. Pearson correlation coefficients of 0.94 to 0.99 were observed between the −Δlog_10_ *K*_*D,app*_ values from two replicates of Tite-Seq experiments, demonstrating the high data quality (S2A and S2B Fig). As a control, we also measured the expression level of each mutation by FACS and next-generation sequencing. Our result showed that most mutations have high expression level, which did not correlate with binding affinity (S2C Fig). Moreover, the binding affinity and expression level of nonsense mutations were distinct from those of silent mutations (S2C Fig), further validating our data.

**Fig 1:**
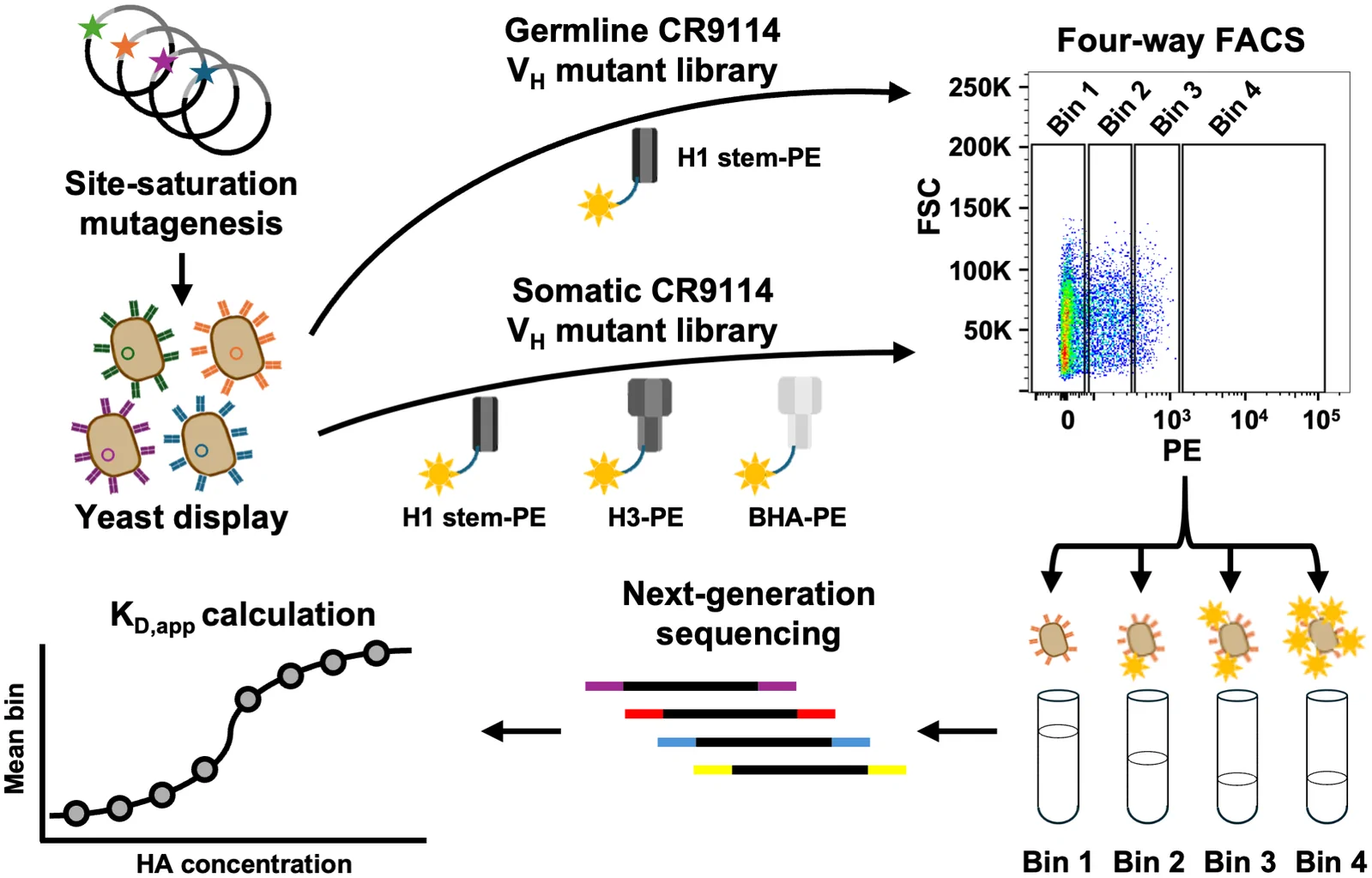
Workflow summary. A schematic representing our Tite-Seq experiments is shown.

**Fig 2:**
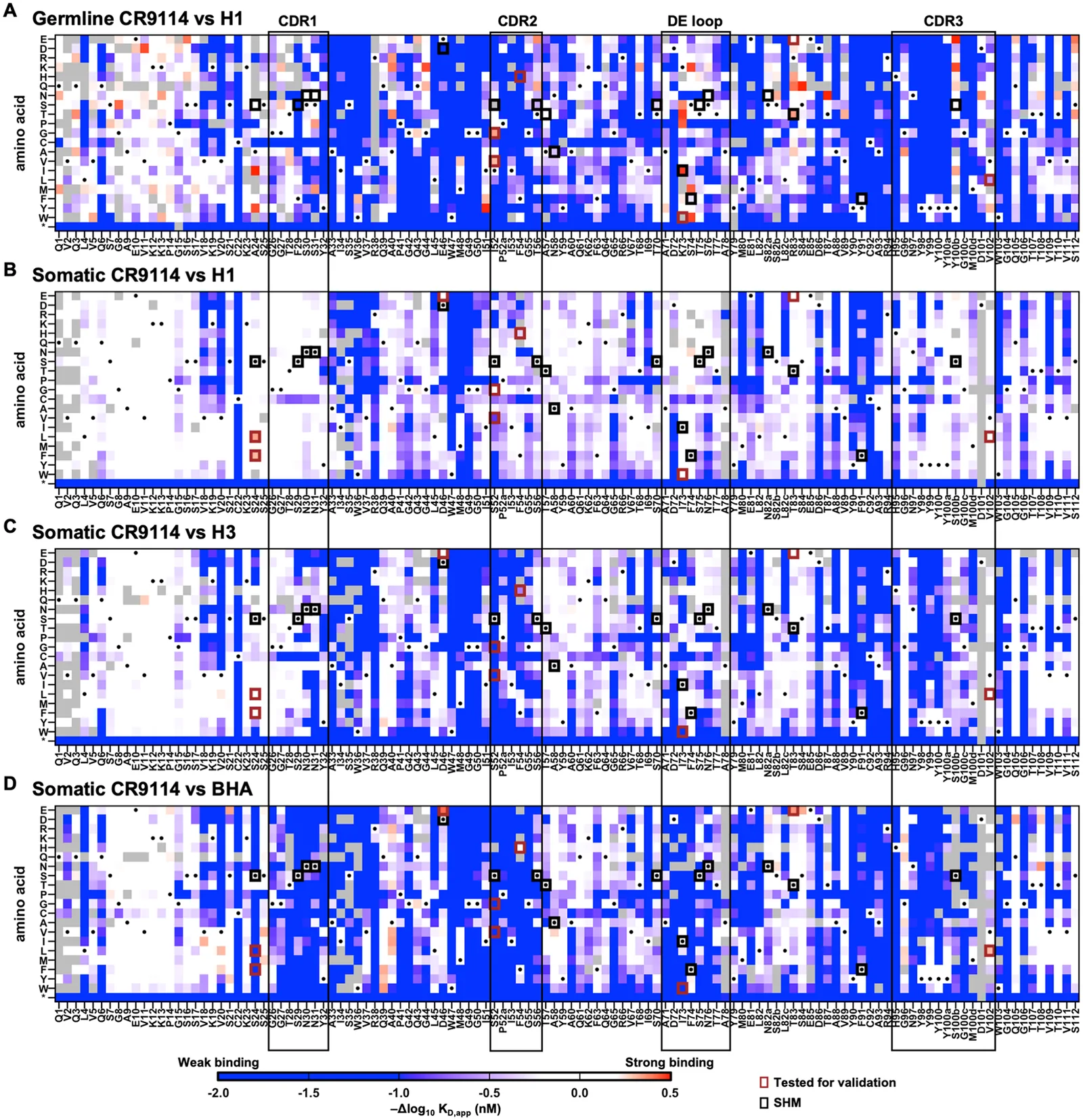
Binding affinity landscapes of germline and somatic CR9114 heavy chain. **(A–D)** Heatmaps showing the −Δlog_10_ *K*_*D,app*_ values of **(A)** single mutations of germline CR9114 against H1 stem, **(B)** single mutations of somatic CR9114 against H1 stem, **(C)** single mutations of somatic CR9114 against H3/Sing16 HA, and **(D)** single mutations of somatic CR9114 against B/Lee40 HA. Gray tiles indicate mutations that were discarded for our analysis due to low occurrence frequency (see Methods). Black rectangles spanning all four heatmaps indicate the locations of the CDRs and DE loop. Black dots indicate the corresponding wild-type sequence (i.e., germline or somatic CR9114). Black squares indicate the somatic hypermutations in CR9114. Brown squares indicate the mutations that were included in our validation experiment. “H1”, “H3”, and “BHA” indicate H1 stem, H3/Sing16 HA, and B/Lee40 HA, respectively. The data underlying this Figure can be found in S1 Data.

A previous study by Phillips and colleagues [16] has performed similar Tite-Seq experiments using a mutant library of CR9114 containing all possible combinations of 16 somatic hypermutations against HAs from H1N1 A/New Caledonia/20/1999, H3N2 A/Wisconsin/67/2005, and B/Malaysia/2506/2004 HA. Because the combinatorial mutant library from Phillips and colleagues [16] contained single amino acid mutants of the germline and somatic CR9114, we were able to compare a small subset of their data to ours. A Pearson correlation coefficient of 0.66 was observed between their −Δlog_10_ *K*_*D,app*_ values and ours (S3A Fig). This correlation coefficient was lower than that between our Tite-Seq experimental replicates, likely due to the different HAs being used for H1 subtype, H3 subtype and influenza B virus. The major difference is that H1 stem was used in our present study, whereas the entire H1 HA ectodomain was used by Phillips and colleagues [16]. Consistently, when we compared only their −Δlog_10_ *K*_*D,app*_ values against H1 HA and ours against H1 stem, the Pearson correlation coefficient decreased to 0.32. Additionally, CR9114 in fragment antigen-binding (Fab) format was used in our present study, whereas single-chain fragment variable (scFv) format was used by Phillips and colleagues [16]. This difference may also affect the impact of a given mutation on binding affinity.

### Mutational tolerance of CR9114 correlates with binding affinity

A previous study demonstrated that the binding affinity of somatic CR9114 to H1 HA was stronger than its affinity to H3 HA, which in turn was stronger than its affinity to BHA [16]. Consistently, our biolayer interferometry (BLI) experiments showed that somatic CR9114 Fab bound to H1 stem, H3/Sing16 HA, and B/Lee40 HA with *K*_*D*_ values of <0.1 nM, 36 nM, and 110 nM, respectively (S4 and S5 Figs). This order of binding affinity of somatic CR9114 against different HAs appeared to correlate with the mutational tolerance, which decreased from H1 stem (median −Δlog_10_ *K*_*D,app*_ = −0.22) to H3/Sing16 HA (median −Δlog_10_ *K*_*D,app*_ = −0.40) to B/Lee40 HA (median −Δlog_10_ *K*_*D,app*_ = −0.82, Figs 2 and S6). Similarly, germline CR9114 had a weaker binding affinity (*K*_*D*_ = 185 nM) and lower mutational tolerance (median −Δlog_10_ *K*_*D,app*_ = −0.85) than somatic CR9114 against H1 stem (Figs 2, S4, and S6). It is well established that proteins with higher thermostability have higher evolvability [19]. Nevertheless, despite the tendency of affinity maturation to decrease antibody thermostability [20], the thermostabilities of germline CR9114 (T_m_ = 77.5 °C) and somatic CR9114 (*T*_m_ = 78.5 °C) were similar (S7 Fig). This observation showed that the difference in mutational tolerance between germline and somatic CR9114 was not due to their thermostability. While it is possible that this difference was due to the stronger binding affinity of somatic CR9114, it is also possible that the mutational stability effect, which we did not measure, was generally less deleterious in somatic CR9114.

### Prevalence of epistasis and pleiotropy in the breadth evolution of CR9114

The binding affinity landscapes of the germline and somatic CR9114 against different HAs allowed us to analyze the involvement of epistasis and pleiotropy in the breadth evolution of CR9114. Here, epistasis refers to how a given mutation has different effects on binding affinity between germline and somatic CR9114, whereas pleiotropy describes how a given mutation affects the binding affinity against different HAs. We first observed that mutations with minimal effects on the binding affinity of germline CR9114 to the H1 stem rarely affected the binding affinity of somatic CR9114 to H1 stem (Figs 3A and S8A), but were frequently detrimental to the binding affinity of somatic CR9114 to H3/Sing16 HA (Figs 3B and S8B), and even more often deleterious to that of B/Lee40 HA (Figs 3C and S8C). These increasingly detrimental mutations throughout the screens mostly resided within the complementarity-determining regions (CDRs), while the effects of mutations in the framework regions on binding affinity correlated well between germline and somatic CR9114 (Figs 3A–3C and S8A–S8C). Our findings indicated that affinity maturation of germline CR9114 against H1 stem could impose a genetic barrier for evolving cross-reactivity to H3 HA and BHA due to epistasis and pleiotropy.

**Fig 3:**
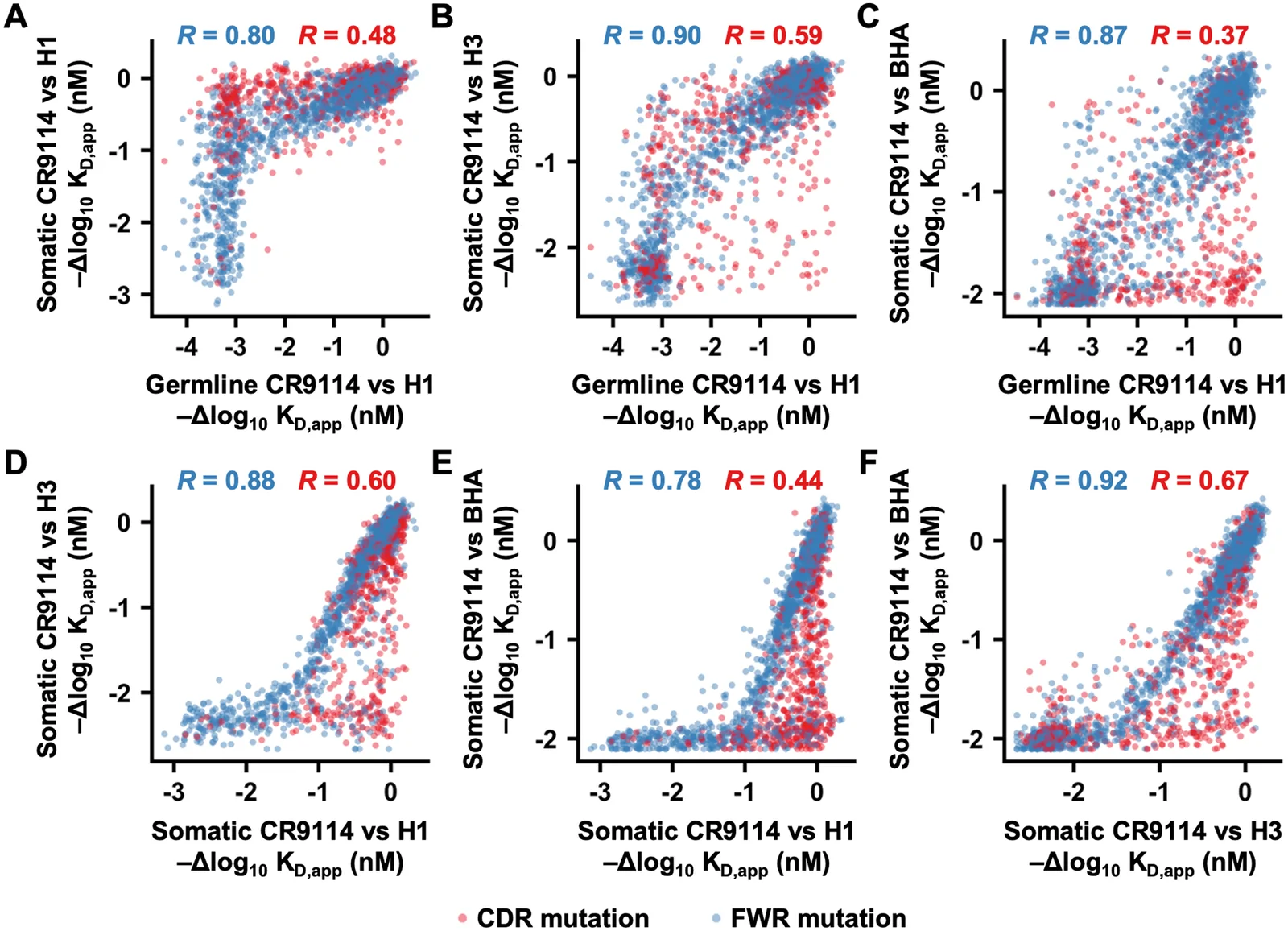
Correlation of mutational effects on binding affinity across different deep mutational scanning experiments. **(A–F)** The −Δlog_10_ *K*_*D,app*_ value of each mutation was compared between the deep mutational scanning experiments of **(A)** the germline CR9114 against H1 stem and the somatic CR9114 against H1 stem, **(B)** the germline CR9114 against H1 stem and the somatic CR9114 against H3/Sing16 HA, **(C)** the germline CR9114 against H1 stem and the somatic CR9114 against B/Lee40 HA, **(D)** the somatic CR9114 against H1 stem and the somatic CR9114 against H3/Sing16 HA, **(E)** the somatic CR9114 against H1 stem and the somatic CR9114 against B/Lee40 HA, **(F)** the somatic CR9114 against H3/Sing16 HA and the somatic CR9114 against B/Lee40 HA. Each data point represents a mutation. Pearson correlation coefficients for the mutations in the CDRs and the DE loop (red) as well as for those in the framework regions (FWRs, blue) are indicated. “H1”, “H3”, and “BHA” indicate H1 stem, H3/Sing16 HA, and B/Lee40 HA, respectively. The data underlying this Figure can be found in S1 Data.

By comparing the binding affinity landscapes of somatic CR9114 against different HAs, we observed a pattern of nested constraint, similar to that described by a previous study on 16 somatic hypermutations of CR9114 [16]. For example, while somatic CR9114 mutations that were detrimental for H1 stem binding were also detrimental for H3/Sing16 HA and B/Lee40 HA binding, many somatic CR9114 mutations that impaired H3/Sing16 HA and B/Lee40 HA binding had minimal effects on H1 stem binding (Figs 3D, 3E, S8D, and S8E). Additionally, while somatic CR9114 mutations that were detrimental for H3/Sing16 HA binding were also detrimental for binding to B/Lee40 HA, many somatic CR9114 mutations that impaired B/Lee40 HA binding had minimal effects on H3/Sing16 HA binding (Figs 3F and S8F). Notably, most mutations that exhibited differential effects on binding against different HAs were in the CDRs (Figs 3D–3F and S8D–S8F). Together, our results suggested that further improving the affinity of somatic CR9114 against H1 HA could result in a loss of cross-reactive breadth due to pleiotropy.

### Epistasis and pleiotropy involve non-paratope mutations

To experimentally validate mutations that exhibited differential effects on the binding affinity of the germline and somatic CR9114 against different HAs, we recombinantly expressed and purified mutants of interest, then measured their binding affinity using BLI (S4 and S5 Figs). The −Δlog_10_ *K*_*D,app*_ values from Tite-Seq had a Pearson correlation coefficient of 0.87 with the −Δlog_10_ *K*_*D*_ values from BLI (Fig 4A). Mutations in this validation experiment included V_H_ S24F and V_H_ S24L, which abolished the binding of somatic CR9114 to B/Lee40 HA, but not to H1 stem and H3/Sing16 HA (Figs 4C, S4, and S5). Our BLI results also showed that V_H_ I52G, V_H_ I52V, and V_H_ K73W had minimal effects on the binding of germline CR9114 to the H1 stem, whereas V_H_ S52G, V_H_ S52V, and V_H_ I73W slightly weakened the binding of somatic CR9114 to H1 stem while completely abolishing its binding to H3/Sing16 HA and B/Lee40 HA (Figs 4C, S4, and S5). The binding affinity of germline and somatic CR9114 mutants against H1 stem also showed strong correlation (Pearson correlation coefficient of 0.85) with their neutralization activity against H1N1 A/Brisbane/59/2007 virus (Fig 4B). Our validation experiment substantiated that epistasis and pleiotropy could constrain the breadth evolution of CR9114.

**Fig 4:**
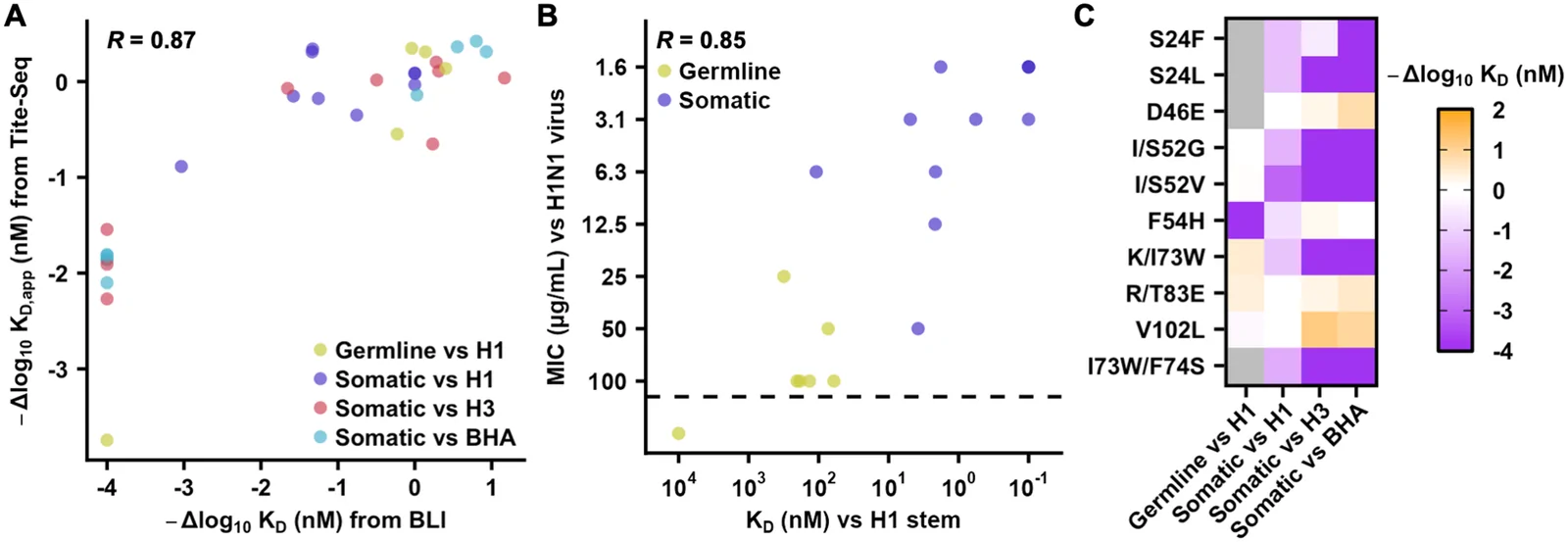
Experimental validation of the deep mutational scanning results. **(A)** The −Δlog_10_ *K*_*D*_ values determined by biolayer interferometry (BLI) are plotted against the −Δlog_10_ *K*_*D,app*_ values determined by Tite-Seq in the deep mutational scanning experiments. Each data point represents a mutation. Mutations with no detectable binding in BLI are assigned a −Δlog_10_ *K*_*D*_ value of −4. The genetic background of each mutation (i.e., germline or somatic CR9114) and the binding target (i.e., H1 stem, H3/Sing16 HA, or B/Lee40 HA) are color-coded. Pearson correlation coefficient (*R*) is indicated. **(B)** The *K*_*D*_ values for mutant binding to H1 stem determined by BLI are plotted against the minimal inhibitory concentration (MIC) values determined by microneutralization assay using H1N1 A/Brisbane/59/2007 virus. Each data point represents a mutation tested on the genetic background (i.e., germline or somatic CR9114) indicated by color. Pearson correlation coefficient (*R*) is indicated. **(C)** Heatmap of BLI results for the indicated mutant in the indicated genetic background (i.e., germline or somatic variant CR9114) against the indicated target (i.e., H1 stem, H3/Sing16 HA, or B/Lee40 HA). “H1”, “H3”, and “BHA” indicate H1 stem, H3/Sing16 HA, and B/Lee40 HA, respectively. The data underlying this Figure can be found in S2 Data.

While V_H_ residue 73 is part of the CR9114 paratope (i.e., antibody region that contacts the epitope), V_H_ residues 24 and 52 are not (Fig 5A). In the somatic CR9114, V_H_ S24 H-bonds with the backbone carbonyl of V_H_ G27 and the side chain of V_H_ S29 to stabilize the conformation of CDR H1 (Fig 5B). Mutations V_H_ S24F and V_H_ S24L would abolish these H-bonds and increase the conformational flexibility of CDR H1. Together with our BLI results (Figs 4C, S4, and S5), this observation suggested that the conformational stability of CDR H1 may be required for somatic CR9114 to bind to BHA, but not to H1 HA and H3 HA. As for V_H_ residue 52, the germline CR9114 had an Ile, whereas the somatic CR9114 had a Ser. In the somatic CR9114, V_H_ S52 H-bonds with the backbone carbonyl of V_H_ Y98 to stabilize the conformation of both CDR H2 and CDR H3 (Fig 5C). However, this H-bond between CDR H2 and CDR H3 would be absent in the germline CR9114 due to its hydrophobic V_H_ I52. As a result, while mutating V_H_ residue 52 in the somatic CR9114 to Gly or Val would abolish an intramolecular H-bond, the same would not occur in the germline CR9114. This observation could explain the differential effects of V_H_ I/S52G and V_H_ I/S52V on the binding affinity of somatic and germline CR9114 (Figs 4C, S4, and S5).

**Fig 5:**
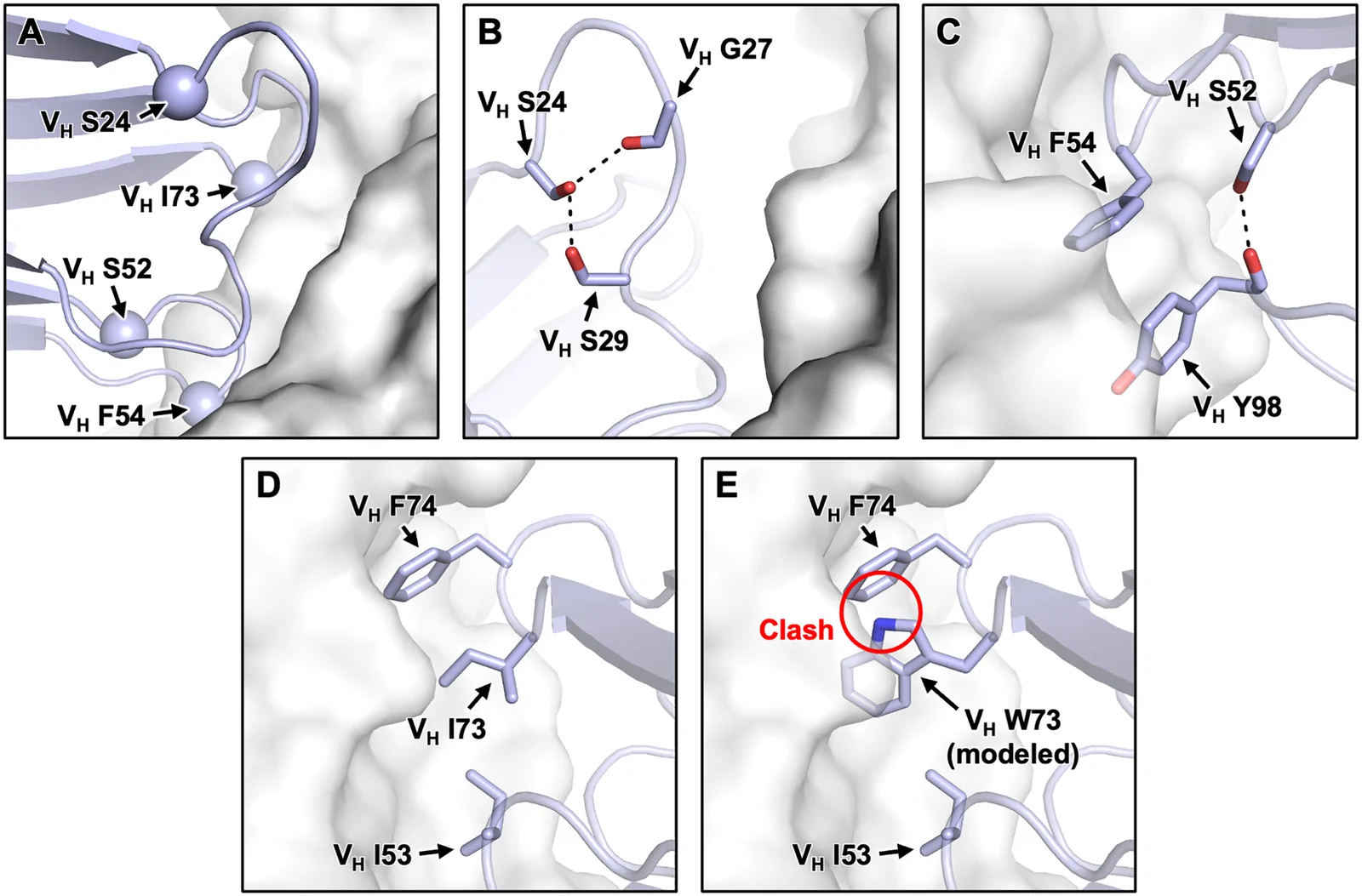
Structural analysis of the differential effects on binding affinity. **(A)** The Cαs of V_H_ S24, V_H_ S52, V_H_ F54, and V_H_ I73 in CR9114 are shown as spheres (PDB 4FQI) [15]. CR9114 and HA are in blue and white, respectively. The local chemical environment for **(B)** V_H_ S24, **(C)** V_H_ S52 and V_H_ F54, and **(D)** V_H_ I73 are shown. Black dashed lines represent H-bonds. **(E)** The structure of HA-bound CR9114 with V_H_ W73 is modeled. Red circle indicates steric clash.

### A paratope mutation constrains the breadth evolution of CR9114

Our BLI results showed that mutating V_H_ residue 73 to Trp improved binding of germline CR9114 H1 stem, but abolished the binding of somatic CR9114 to H3/Sing16 HA and B/Lee40 HA (Figs 4C, S4, and S5). V_H_ residue 73 is part of the CR9114 paratope. In somatic CR9114, V_H_ I73 is adjacent to V_H_ F74 (Fig 5D). Our structural modeling indicated that mutation V_H_ I73W would introduce a steric clash with V_H_ F74 (Fig 5E), which could explain its detrimental effect on the binding of somatic CR9114 to H3/Sing16 HA and B/Lee40 HA. By contrast, this detrimental steric clash was not observed in the germline CR9114, which has the small amino acid Ser at V_H_ residue 74 (Fig 2). However, somatic CR9114 with double mutations V_H_ I73W/F74S was still unable to bind to H3/Sing16 HA or B/Lee40 HA (Figs 4C and S5). Therefore, V_H_ W73 could impose strong constraints for CR9114 to evolve breadth, which may not be easily overcome by a secondary mutation.

### Epistasis is prevalent in IGHV1-69 HA stem antibodies

CR9114 is encoded by IGHV1-69, which is a recurring sequencing feature of HA stem antibodies [21–23]. Our previous study has curated over 5,000 influenza virus HA antibodies from the literature, of which 160 are IGHV1-69 HA stem antibodies [24]. Here, we analyzed the somatic hypermutations present in these 160 IGHV1-69 HA stem antibodies in terms of their effects on the binding affinity of germline CR9114. Most of the IGHV1-69 HA stem antibodies contained at least one somatic hypermutation with a −Δlog_10_ *K*_*D,app*_ value of less than −1 (Fig 6), which was a stringent cutoff for deleterious mutations according to our validation experiment (Fig 4A). These observations suggested that epistasis is prevalent among the affinity maturation trajectories of IGHV1-69 HA stem antibodies. This epistasis could be due to a given mutation having different effects on the binding affinity of the germline precursors of different IGHV1-69 HA stem antibodies. Consistently, different binding modes have been reported for IGHV1-69 HA stem antibodies [22,25]. Another possibility is that a given mutation may be detrimental to the germline precursors of all IGHV1-69 HA stem antibodies, but may become neutral or beneficial in the presence of other somatic hypermutations, as observed in CR9114 [16].

**Fig 6:**
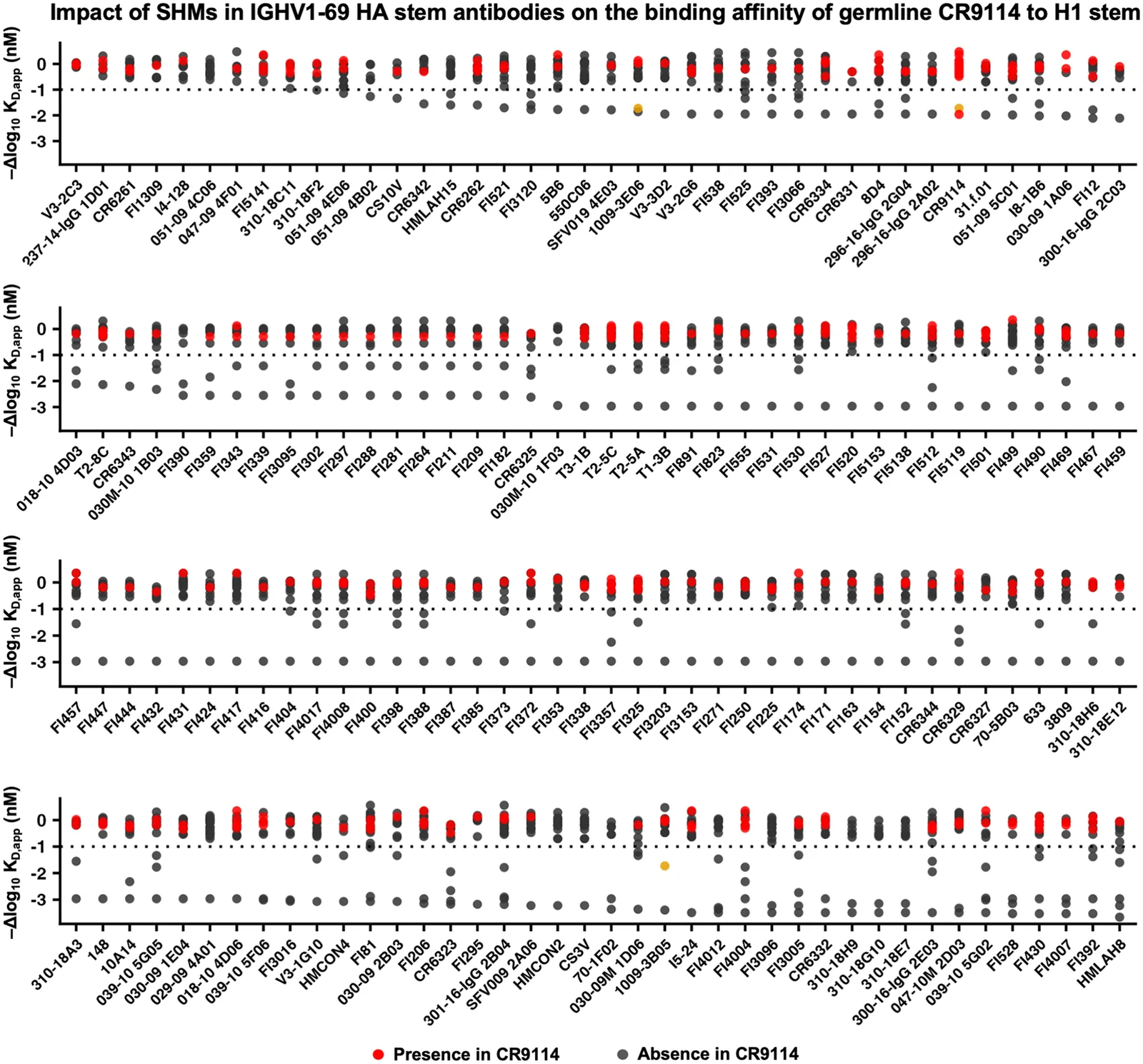
The effects of somatic hypermutations in IGHV1-69 HA stem antibodies on the binding affinity of germline CR9114 to H1 stem. The somatic hypermutations in each of the 160 known IGHV1-69 HA stem antibodies [24] were identified. Their corresponding −Δlog_10_ *K*_*D,app*_ values obtained from the deep mutational scanning experiment of the germline CR9114 against H1 stem (Fig 2) are shown. Each data point represents one somatic hypermutation of the indicated antibody. Somatic hypermutations observed in CR9114 are in red, except that V_H_ F29S is in mustard. The name of each antibody is shown on the x-axis. The data underlying this Figure can be found in S3 Data.

To further examine the presence of epistasis in another IGHV1-69 HA stem antibody, we focused on the somatic hypermutation V_H_ S35N in 310-18G10, which was previously isolated from an H5N1 vaccinee [26]. V_H_ S35N is highly deleterious for both germline and somatic CR9114 (Fig 2), yet it is present in 8% of IGHV1-69 HA stem antibodies (S9 Fig), including 310-18G10. The binding affinity of the inferred germline of 310-18G10 (hereinafter referred to as germline 310-18G10) to H1 stem was weakened by 15% upon the addition of V_H_ S35N (S10 Fig). At the same time, reverting V_H_ N35 to V_H_ S35 weakened the binding affinity of somatic 310-18G10 to H1 stem by almost 4-fold. Therefore, V_H_ S35N contributed to the affinity maturation of 310-18G10, despite being deleterious in the germline 310-18G10. These results further substantiated the present of epistasis among the binding affinity landscapes of IGHV1-69 HA stem antibodies.

As a somatic hypermutation in CR9114, V_H_ F29S has previously been shown to have a negative first-order effect on CR9114 binding to H1N1 A/New Caledonia/1999 HA, but it has positive epistatic interactions with V_H_ I52S, V_H_ K73I, V_H_ S74F, V_H_ T75S, and V_H_ S76N [16]. Similarly, we observed that V_H_ F29S was highly deleterious for the binding of germline CR9114 to H1 stem (Fig 2). We found that V_H_ F29S was present in two other IGHV1-69 HA stem antibodies, namely 1009-3B05 and 1009-3E06 (Figs 6 and S11). Notably, both 1009-3B05 and 1009-3E06 were isolated from the same influenza patient and belong to the same clonotype [24,27]. While 1009-3E06 contained one of the known compensatory mutations, V_H_ S76N, 1009-3B05 did not contain any (S11 Fig). This observation suggested that an unknown epistatic interaction involving V_H_ F29S was present in 1009-3B05.

## Discussion

CR9114 was one of the first few bnAbs discovered against influenza virus HA stem [15]. Given that CR9114 is encoded by the commonly used IGHV1–69 germline gene and lacks unusual features that are observed in bnAbs to human immunodeficiency virus type 1 (HIV-1), such as extensive somatic hypermutation [28], long indels [29], or domain swapping [30], CR9114-like antibodies were initially thought to be readily generable and present in different individuals [15]. However, despite being discovered more than a decade ago, CR9114 remains the only bnAb found to date that cross-reacts with both influenza A and B virus HAs. In fact, most IGHV1-69 HA stem antibodies are specific to group 1 HA and cannot even cross-react with group 2 HA [24,31]. To understand the potential evolutionary constraints that would limit the elicitation of CR9114-like antibodies, this study systematically analyzed the mutational effects on the binding affinity of the germline and somatic CR9114 against multiple HAs. We found that the breadth evolution of CR9114 is highly vulnerable due to epistasis and pleiotropy. Our results not only help explain the rarity of HA stem bnAbs that cross-react with influenza A and B viruses, but also have important implications for developing broadly protective influenza vaccines.

A notable finding in this study was that the mutational tolerance of somatic CR9114 decreased in correlation with its binding affinity, which weakened sequentially across the H1 stem, H3 HA, and BHA. This observation is substantiated by our validation experiment, where mutations V_H_ S52G, V_H_ S52V, and V_H_ I73W abolished the binding of somatic CR9114 to H3 HA and BHA, but not to H1 stem. Consistently, the mutational tolerance and binding affinity of germline CR9114 against H1 stem were similar to those of somatic CR9114 against BHA. This phenomenon parallels a previous study which showed the presence of sequence determinants that are functionally redundant for H1 HA binding in somatic but not germline IGHV1-69 HA stem antibodies [21]. However, the underlying biophysical mechanisms and its generalizability to other classes of antibodies warrant additional studies.

Phillips and colleagues have previously shown that the breadth evolution of CR9114 follows a sequential gain-of-affinity in the order of H1 HA, H3 HA, then BHA, due to nested evolutionary constraints also observed in our study here [16]. Nevertheless, a sequential vaccination scheme for reliably eliciting CR9114-like bnAbs may be convoluted, because of the large number of potential mutations that are detrimental to H3 HA and BHA binding without affecting H1 HA binding. As a result, even if CR9114-like bnAb precursors can be primed by H1 HA, they might acquire somatic hypermutations that prevent the subsequent acquisition of cross-reactivity breadth, especially since selection in the germinal centers is weak [32]. At the same time, multiple somatic hypermutations may occur simultaneously. A recent study has further shown that secondary compensatory mutations can occur before an initial deleterious somatic hypermutation is purged from the germinal centers [33]. Given that multiple somatic hypermutations are required for CR9114 to acquire cross-reactivity breadth [16], it is possible that CR9114-like bnAbs can develop through alternative evolutionary trajectories involving epistatic mutations not captured in our present study. However, compared to the difficulties in eliciting CR9114-like bnAbs that cross-react with influenza A and B virus HAs, the generation of HA stem bnAbs with pan-influenza A virus HA reactivity appeared much easier [34–36]. For example, a single amino acid mutation is able to confer cross-group reactivity to the multidonor HA stem bnAbs encoded by IGHV1-18 with a QxxV motif in the CDR H3 [34]. Therefore, we postulate that a reasonable strategy for developing broadly protective influenza vaccines against both human and zoonotic strains is to use one immunogen to elicit pan-influenza A virus bnAbs and a second immunogen for pan-influenza B virus bnAbs. Notably, although only a handful of monoclonal antibodies targeting the influenza B virus HA stem have been identified [37–39], human antibody responses to the influenza B virus HA stem are prevalent and may confer protection [40,41].

To date, multiple HA-stem based influenza vaccine candidates have been developed [12,18,42–45]. Nevertheless, both group 1 and group 2 HA stem-based vaccine candidates tested in clinical studies have shown limited ability to elicit cross-group bnAbs, despite robustly eliciting group-specific bnAbs [46–49]. The lack of a reliable method for eliciting cross-group HA stem bnAbs represents a major obstacle toward a pan-influenza A virus vaccine. At the same time, a recent study has demonstrated that the antibody evolutionary outcomes in germinal centers are predictable based on the antibody binding affinity landscapes and somatic hypermutation biases [32]. Therefore, continued characterization of the binding affinity landscapes of diverse HA stem bnAbs will be crucial for guiding the rational development of a pan-influenza A virus vaccine.

## Methods

### Yeast display plasmids

Yeast display plasmids for germline and somatic CR9114 Fabs were constructed based on the pCTcon2 vector, with an open reading frame encoding (from N-terminal to C-terminal): Aga2 secretion signal, somatic CR9114 Fab light chain, V5 tag, equine rhinitis B virus (ERBV-1) 2A peptide, Aga2 secretion signal, germline or somatic CR9114 Fab heavy chain, HA-tag, and Aga2p.

### Construction of yeast Fab mutant libraries

Two separate mutant libraries were generated based on different versions of the CR9114 heavy chain variable domain sequence. The fully developed or somatic CR9114 sequence, and the pre-developmental, or germline CR9114 sequence, were each used as bases for the respective libraries. The heavy and light chain sequences of somatic CR9114 were obtained from Genbank JX213639 and JX213640, respectively [15]. The CR9114 germline sequence was obtained from Phillips and colleagues [16], who themselves had reconstructed the sequence through the international ImMunoGeneTics information system (IMGT) [50] and IgBLAST [51]. We chose to mimic their treatment of site V_H_ S109N, and not acknowledge a mutation at that site [16]. For both libraries, the somatic CR9114 light chain sequence was used, to focus solely on heavy chain variant effects. The mutant libraries of variant Fabs were generated that differed from the somatic or germline CR9114 sequences by only one amino acid per mutant, such that every possible amino acid, as well as stop codons, was represented in every possible position of the heavy chain variable domain, excluding the final amino acid in the heavy chain variable domain, which is highly conserved.

The pCTcon2 plasmid containing either the somatic or germline CR9114 sequence in Fab form was used as a template to generate the respective mutant library inserts and the linearized vector through polymerase chain reaction (PCR), separately. The inserts for each library were then generated as follows, per library. To generate the linearized vectors, we used 5′-TGG TGT CCA CAC TTT CCC TGC TGT T-3′ and 5′-AGT GGA TTG GGG ATT GGC TTT CCG C-3′ as primers. To generate the full-length inserts, we first needed to manufacture two sets of 15 split inserts per library in parallel before merging them in paired PCRs using overlap extension PCR. The first set of split inserts included 15 separate reactions; each performed with a cassette of pooled forward primers and a universal reverse primer 5′-AGG AGT ACA AAC CGG AAG ATT GC-3′. Each forward cassette of primers was composed of eight forward primers in equal molar ratios with an identical 21 nt at the 5′ end and 15 nt at the 3′ end. NNK (N: A, C, G, T; K: G, T) sequences were present within each cassette, placed in such a way that saturation mutagenesis was achieved. As described previously, silent (or synonymous) mutations were introduced into each primer to discern between sequencing errors and target mutations through data analysis [52]. In S1 Table, the forward primers are listed for each mutant library, identified in the table by cassette number and the position of the amino acid mutated. The second set of split inserts also included 15 separate reactions, designed to match up with the first set of split inserts. This final set of 15 insert fragments was generated through PCR, with each reaction using the universal forward primer 5′-ACT GTC GCC CCA ACT GAG TGC TC-3′ and a unique reverse primer (S2 Table). The first and second sets of split inserts were joined to generate full-length cassettes through 15 overlapping PCRs, using universal forward and reverse primers to perform the amplifications with 10 ng of the respective inserts per PCR. For each library, these 15 full-length cassettes were gel purified, then mixed using equimolar amounts of each cassette to generate the final insert pool for use in the yeast transformation. PrimeSTAR Max polymerase (Takara Bio, cat no. R045B) was used for all PCRs. Gel purification was performed using the Monarch Gel Extraction Kit (New England Biolabs, cat no. T1020L). The manufacturer’s instructions were used for all samples in each case.

### Yeast transformation

Transformation of yeast was performed for each mutant library by electroporation as previously described [53]. Strain EBY100 of *Saccharomyces cerevisiae* (ATCC) were grown overnight at 30 °C with 250 rpm agitation in YPD medium (1% w/v yeast nitrogen base, 2% w/v peptone, 2% w/v D(+)-glucose). Once the culture reached OD_600_ of 3, a new culture was seeded at OD_600_ of 0.3 in 100 mL of YPD media and continued to grow at 30 °C with 250 rpm agitation. Following OD_600_ reaching 1.6, the culture of yeast cells was centrifuged for 3 min at 1,700*g* at room temperature, and the media was removed. Fifty milliliters of ice-cold water was used to wash the cell pellet twice; the pellet was then washed with 50 mL of ice-cold electroporation buffer (1 M sorbitol, 1 mM calcium chloride) before resuspension in 20 mL conditioning media (0.1 M lithium acetate, 10 mM dithiothreitol). The new mixture was then shaken at 250 rpm at 30 °C for 30 min, then centrifuged at 1,700*g* for 3 min at room temperature. Following wash with 50 mL ice-cold electroporation buffer, the pellet was resuspended in 1 mL of electroporation buffer and placed on ice. In parallel, 4 µg of the linearized vector was mixed with 5 µg of the full-length cassette mutant library insert, and pipetted into 400 µL of the prepared 1 mL conditioned yeast in a pre-chilled BioRad GenePulser 2 mm electrode gap cuvette. Following a 5-min incubation on ice, electroporation was performed at 2.5 kV, 25 µF, such that the time constant was within 3.7 and 4.1 ms. The electroporated cell mixture was immediately transferred into 8 mL of a 1 to 1 mixture of YPD and 1 M sorbitol, then shaken at 30 °C, 250 rpm for 1 h.

Cells were collected via centrifugation at 1,700*g* for 3 min at room temperature, resuspended in 0.6 mL synthetic dextrose casamino acid medium (SD-CAA: 2% w/v D-glucose, 0.67% w/v yeast nitrogen base with ammonium sulfate, 0.5% w/v casamino acids, 0.54% w/v Na_2_HPO_4_, 0.86% w/v NaH_2_PO_4_·H_2_O, all dissolved in deionized water), plated onto SD-CAA plates (2% w/v D-glucose, 0.67% w/v yeast nitrogen base with ammonium sulfate, 0.5% w/v casamino acids, 0.54% w/v Na_2_HPO_4_, 0.86% w/v NaH_2_PO_4_·H_2_O, 18.2% w/v sorbitol, 1.5% w/v agar, all dissolved in deionized water) and incubated at 30 °C for 48 h. Colonies were then collected in SD-CAA medium, centrifuged at 1,700*g* for 5 min at room temperature, and resuspended in SD-CAA medium with 15% v/v glycerol such that OD600 was 50. Glycerol stocks were stored at −80 °C until used.

### Cell culture

Sf9 cells (*Spodoptera frugiperda* ovarian cells, female, ATCC, cat no. CRL-1711) were maintained in Sf-900 II SFM medium (Thermo Fisher Scientific, cat no. 10902088) at 37 °C. Expi293F cells (Thermo Fisher Scientific, cat no. A14527) were grown and maintained in Expi293 Expression Medium (Thermo Fisher Scientific, cat no. A1435101) at 37 °C, 8% CO_2_, and 95% humidity with shaking at 125 rpm according to the manufacturer’s instructions. Madin-Darby canine kidney cells stably transfected with the cDNA of human 2,6-sialyltransferase (MDCK-SIAT1) (ATCC, cat no. CRL-3743) were grown and maintained in Dulbecco’s Modified Eagle’s Medium (Thermo Fisher Scientific, cat no. 11995073) containing 10% fetal bovine serum (Fisher Scientific, cat no. A5669701), 1× GlutaMAX supplement (Thermo Fisher Scientific, cat no. 35050079), and 100 U mL^−1^ penicillin/streptomycin (Thermo Fisher Scientific, cat no. 15140122) at 37 °C, 5% CO_2_, and 95% humidity, according to the manufacturer’s instructions.

### Influenza virus

Recombinant 6:2 reassortant virus with hemagglutinin (HA) and neuraminidase (NA) segments from H1N1 A/Brisbane/59/2007 in H1N1 A/Puerto Rico/8/1934 (PR8) backbone were rescued using the eight-plasmid influenza virus reverse genetics system [54]. Briefly, DNA plasmids were transfected into a co-culture of HEK293T cells and MDCK-SIAT1 cells (ratio of 6:1) and incubated at 37 °C for 48 h. Supernatants were then injected into 8- to 10-day-old embryonated chicken eggs for viral rescue at 37 °C for 48 h. Virus was plaque-purified on MDCK-SIAT1 cells. Individual plaques were picked and injected into embryonated eggs, and viral RNAs were extracted from allantoic fluids. HA and NA segments were confirmed by Sanger sequencing.

### Expression and purification of H1 stem, H3 HA, and BHA

Oligonucleotide encoding H1 stem #4900 protein [18], H3 HA (A/Singapore/INFIMH-16-0019/2016; GISAID accession num. EPI1858150), or BHA (B/Lee/1940; GISAID accession num. EPI243230) was fused with N-terminal gp67 signal peptide and a C-terminal BirA biotinylation site, thrombin cleavage site, T4-foldon trimerization domain, and a 6× His tag, and then cloned into a customized baculovirus transfer vector [55]. Recombinant bacmid DNA that carried the protein construct was generated using the Bac-to-Bac system (Thermo Fisher Scientific, cat no. 10359016) according to the manufacturer’s instructions. Baculovirus was generated by transfecting the purified bacmid DNA into adherent Sf9 cells using Cellfectin reagent (Thermo Fisher Scientific, cat no. 10362100) according to the manufacturer’s instructions. The baculovirus was further amplified by passaging in adherent Sf9 cells at a multiplicity of infection (MOI) of 1. Recombinant protein was expressed by infecting 1 L of suspension Sf9 cells at an MOI of 1. Infected Sf9 cells were incubated at 27 °C with shaking at 170 rpm for 72 h. On day 3 post-infection, Sf9 cells were pelleted by centrifugation at 4,000*g* for 25 min, and soluble recombinant protein was purified from the supernatant by affinity chromatography using Ni Sepharose excel resin (Cytiva, cat no. 17371202) and then size exclusion chromatography using a HiLoad 16/100 Superdex 200 prep grade column (Cytiva, cat no. 90100137) in 20 mM Tris-HCl pH 8.0, 100 mM NaCl. The purified protein was concentrated by Amicon spin filter (Millipore Sigma, cat no. UFC901008) and filtered by 0.22 mm centrifuge Tube Filters (Costar, cat no. 8160). Concentration of the protein was determined by a NanoDrop One^C^ spectrophotometer (Fisher Scientific). Protein was subsequently aliquoted, flash frozen in dry-ice ethanol mixture, and stored at −80 °C until use.

### Biotinylation and PE-conjugation of HAs

Purified H1 stem, H3 HA, and BHA were biotinylated using the Biotin-Protein Ligase-BIRA kit according to the manufacturer’s instructions (Avidity, cat no. BirA500). After buffer exchange into phosphate-buffered saline (PBS), the biotinylated HA were conjugated to streptavidin-PE (Fisher Scientific, cat no. S866A) by incubating at room temperature for 15 min.

### Fluorescence-activated cell sorting (FACS) of yeast display libraries

Hundred microliters glycerol stock of the yeast display library was recovered in 50 mL SD-CAA medium by incubating at 27 °C with shaking at 250 rpm until OD_600_ reached between 1.5 and 2.0. Then 15 mL of the yeast culture was harvested and pelleted via centrifugation at 4,000*g* at 4 °C for 5 min. The supernatant was discarded, and sucrose/galactose/raffinose/casamino acids medium (SGR-CAA: 2% w/v galactose, 2% w/v raffinose, 0.1% w/v D-glucose, 0.67% w/v yeast nitrogen base with ammonium sulfate, 0.5% w/v casamino acids, 0.54% w/v Na_2_HPO_4_, 0.86% w/v NaH_2_PO_4_·H_2_O, all dissolved in deionized water) was added to make up the volume to 50 mL. The yeast culture was then transferred to a baffled flask and incubated at 18 °C with shaking at 250 rpm. Once OD_600_ reached between 1.3 and 1.6, 1 mL of yeast culture was harvested and pelleted via centrifugation at 4,000*g* at 4 °C for 5 min. The pellet was subsequently washed with 1 mL of 1× PBS twice. After the final wash, cells were resuspended in 1 mL of 1× PBS. For each expression sort, PE anti-HA.11 (epitope 16B12, BioLegend, cat. no. 901517) that had been buffer-exchanged into 1× PBS was added to the cells at a final concentration of 30 nM.

For the expression sorts, PE anti-HA.11 (BioLegend, cat no. 901518) was added to a subset of the washed cells at a final concentration of 30 nM. For Tite-Seq, cells were labeled with PE-conjugated H1 stem, H3 HA, or BHA at each of eight antigen concentrations. For the somatic CR9114 mutant library sorts, one-log increments of PE-conjugated probe spanning 0.01 nM–10 nM and 0.003 nM–3 nM (H1 stem); 0.1 nM–100 nM and 0.03 nM–33 nM (H3 HA); or 0.2 nM–200 nM and 0.067 nM–67 nM (BHA) were used for FACS. For the germline CR9114 mutant library sorts, one-log increments of PE-conjugated H1 stem spanning 16 nM–0.016 nM and 5 nM–0.005 nM, along with an unstained sample, were used for FACS. A negative control was set up with no probe added to the resuspended cells. For the somatic CR9114 mutant library sorts, all cells were stained in solution containing 1× PBS with 1% bovine serum albumin (BSA), while for the germline CR9114 mutant library sorts, all cells were stained in solution of 1× PBS lacking BSA. The reason for this discrepancy is that the method was improved during this project: the addition of 1% BSA was found to provide a cleaner sort with lower background signal. Samples were incubated with their respective probe concentrations for 1 h at 4 °C with rotation. Then the yeast pellet was washed twice in 1× PBS and resuspended in FACS tubes containing 2 mL 1× PBS. Cells in the selected gates were collected in 1 mL of SD-CAA containing 100 U mL^−1^ penicillin/streptomycin (ThermoFisher Scientific, cat no. 15140122). The somatic CR9114 mutant library was sorted using a BD FACSMelody Cell Sorter and BD FACSChorus Software (BD Biosciences), while the germline CR9114 mutant library was sorted using a BD FACSAria and BD FACSDiva Software (BD Biosciences).

While we used a 1-h incubation time for probe staining, longer incubation times were also tested as part of our pilot experiments. Briefly, each yeast display library was incubated with different concentrations of PE-conjugated probes. After incubation for either 1 h, 2 h, or 24 h, samples were measured by a NovoCyte Opteon (Agilent). We saw no obvious difference in binding trends after extending the incubation time for more than 1 h (S12A Fig). Moreover, the MFI of the 1-h probe incubation was similar to that of the 24-h incubation, indicating that binding was close to equilibrium, if not already reached, after 1 h. To measure probe depletion effects, a pre-depletion sample set was prepared by incubating yeast mutant library samples with 0.1–200 nM of PE-conjugated probe for 1 h, then pelleting the yeast by centrifuging at 4,000*g* at 4 °C for 5 min. To prepare the post-depletion samples, the probe-containing supernatants were removed from the pre-depletion sample yeast pellets. This probe-containing ‘depleted’ supernatant was applied to an equivalent number of yeast cells for 1 h to generate the post-depletion sample set. Both pre- and post-depletion samples were analyzed by a NovoCyte Opteon (Agilent). The result showed that there was no probe depletion at all the tested concentrations (S12B Fig).

For expression sort, single cells were gated into four equally populated bins, with bin 1–4 having the increasing PE signal. For Tite-Seq, single cells were gated into four bins along the PE-A axis based on unstained and CR9114 controls, with bin 1 comprising all PE-negative cells, bin 4 comprising PE-positive cells with comparable expression or binding affinity to the germline or somatic CR9114 positive population, and bins 2 and 3 splitting the intermediate population between bin 1 and bin 4. Following sort, the sort tubes were directly placed at 30 °C with 250 rpm agitation. After 20–40 h, the cells in each tube were collected by centrifugation at 3,800*g* for 15 min once a large pellet was visible or the culture was cloudy. Frozen stocks were made per sorted sample by reconstituting the pellet in 15% v/v glycerol (in SD-CAA medium) and then stored at −80 °C until use.

### Next-generation sequencing of FACS samples

Plasmids from the yeast cells before and after sorting were extracted using a Zymoprep Yeast Plasmid Miniprep II Kit (Zymo Research, cat no. D2004) following the manufacturer’s protocol. The region of interest in the mutant library was subsequently amplified by PCR using primers 5′-C ACT CTT TCC CTA CAC GAC GCT CTT CCG ATC TGT GTA CTT GCC GCC GCT CAA CCA −3′ and 5′-G ACT GGA GTT CAG ACG TGT GCT CTT CCG ATC TAC GGA AGG TCC CTT AGT AGA AGC-3′ for both the germline and somatic mutant libraries. Subsequently, adapters containing sequencing barcodes were appended to the amplicon using primers 5′-AAT GAT ACG GCG ACC ACC GAG ATC TAC ACX XXX XXX XAC ACT CTT TCC CTA CAC GAC GCT-3′, and 5′-CAA GCA GAA GAC GGC ATA CGA GAT XXX XXX XXG TGA CTG GAG TTC AGA CGT GTG CT-3′. Positions annotated by an ‘‘X’’ represented the nucleotides for the index sequence. All PCRs post-FACS were performed using Q5 High-Fidelity DNA polymerase (NEB, cat no. M0492S) according to the manufacturer’s instructions. PCR products were purified using PureLink PCR Purification Kit (Thermo Fisher Scientific, cat no. K310002). The final PCR products were submitted for next-generation sequencing using NovaSeq SP PE250 (Illumina).

### Computing the binding score, expression score, and apparent *K*_*D*_ values

The sequencing data were initially obtained in FASTQ format, then analyzed using a custom Python Snakemake pipeline [56]. Briefly, Paired-End reAd mergeR (PEAR) was used to merge the forward and reverse reads [57]. The number of reads corresponding to each variant in each sample was counted. To avoid division by zero in downstream analysis, a pseudocount of 1 was added to the final count.

For each expression sort, the enrichment value of each mutation mut were computed as follows:


$$\begin{array}{c} enrichment(var)=\;\frac{\sum \left({freq}_{i}(mut)\times {MFI}_{i}\right)}{\sum {freq}_{i}(mut)} \end{array}$$

where the ${freq}_{i}(var)$ is the occurrence frequency of mutation mut in bin i and ${MFI}_{i}$ is the mean fluorescence intensity of bin i. The expression score for each variant var was further computed from the enrichment value as follows:


$$\begin{array}{c} Expression\;score(mut)=\frac{enrichment(mut)-\overset{―}{{enrichment}_{nonsense}}}{{enrichment}_{silent}-\overset{―}{{enrichment}_{nonsense}}} \end{array}$$

where $\overset{―}{{enrichment}_{silent}}$ is the average enrichment value of the silent mutations and $\overset{―}{{enrichment}_{nonsense}}$ is the average enrichment value of the nonsense mutations. The final score for each variant *var* is the average of two biological replicates.

To compute apparent $K_{D}$ value of each variant from the Tite-Seq data, we adopted the analysis approach as previously described [58]. Briefly, to determine the mean bin of PE fluorescence for each variant var at each HA concentration, a simple weighted mean calculation was applied:


$$\begin{array}{c} \overset{―}{{Bin}_{\left[HA\right]}}(mut)=(\frac{\sum_{i=\text{0}}^{\text{2}}N_{i,[HA]}(mut)*(i))}{(\sum_{i=\text{0}}^{\text{2}}N_{i,[HA]}}(mut)) \end{array}$$

where $N_{i,[HA]}(mut)$ is the number of cells with mutation mut that fall into bin i at HA concentration [HA]. This calculation computes a weighted average by assigning integer weight to the bin i.

For each variant *var*, we estimated its sorted cell count $N_{i,[HA]}(mut)$ that corresponds to bin i at HA concentration [HA] as follows:


$$\begin{array}{c} N_{i,[HA]}(mut)={Ntotal}_{i,[HA]}\times \frac{C_{i,[HA]}(mut)}{{Ctotal}_{i,[HA]}} \end{array}$$

where variant read count $C_{i,[HA]}(mut)$ is the read counts for variant *var* in bin i at HA concentration [HA], ${Ctotal}_{i,[HA]}$ is the total read counts for bin i at HA concentration [HA], ${Ntotal}_{i,[HA]}$ is the total number of cells in bin i at HA concentration [HA]. We then determined the apparent binding affinity $K_{D,app}$ value for each mutation $K_{D,app}(mut)$ via a nonlinear least-squares regression using a standard non-cooperative Hill equation:


$$\begin{array}{c} \overset{―}{{Bin}_{\left[HA\right]}}(mut)=a\times \frac{\left[HA\right]}{\left(K_{D,app}(mut)+[HA]\right)}+b \end{array}$$

where free parameters *a* is titration response range and *b* is titration curve baseline. Mutations with an occurrence frequency below 0.01% in our pre-sort samples were discarded from our downstream analysis. To quantify the effects of each mutation on binding affinity, we calculate the difference between the $K_{D,app}$ values of the given mutation and the corresponding wild type:


$$\begin{array}{c} -\Delta {\text{log}}_{\text{10}}K_{D,app}(mut)={\text{log}}_{\text{10}}K_{D,app}(WT)-{\text{log}}_{\text{10}}K_{D,app}(mut) \end{array}$$

For this analysis, ligand depletion effects were not considered because probe depletion was not observed in a pilot experiment (S12B Fig).

Of note, while the log_10_ *K*_*D,app*_ values (not Δlog_10_ *K*_*D,app*_ values) from Tite-Seq correlated well with the *K*_*D*_ values from the BLI experiment (Pearson correlation coefficients of 0.79), the log_10_ *K*_*D,app*_ values were systematically lower than the log_10_ *K*_*D*_ values for germline CR9114 mutants against H1 stem (below the diagonal line in S3B Fig). Similarly, while germline CR9114 is known to have a much weaker binding affinity than somatic CR9114 to H1 stem (S4 Fig) [16,59], the *K*_*D,app*_ value for germline CR9114 against H1 stem was 0.3 nM, which was similar to that for somatic CR9114 against H1 stem 0.7 nM. These observations indicated that the Tite-Seq experiment for germline CR9114 mutant library against H1 stem overestimated the binding affinity. One possible explanation for this artifact is that the PE-conjugated probe for the Tite-Seq experiment of the germline CR9114 mutant library against the H1 stem was prepared much earlier than the ones for other Tite-Seq experiments in this study, including that for the somatic CR9114 mutant library against the H1 stem. There may have been a batch effect in the preparation of the PE-conjugated probe, which introduced systematic noise into the *K*_*D,app*_ value measurement.

### Expression and purification of antibodies

The heavy and light chains of CR9114 or 310-18G10 fragment antigen-binding (Fab) or immunoglobulin G (IgG) germline or somatic heavy chain sequence mutants were cloned into phCMV3 vector. The mutants were generated using mismatch PCR to modify the bases desired using PrimeSTAR Max polymerase (Takara Bio, cat no. R045B). The heavy and light chain sequences of somatic 310-18G10 were obtained from GenBank KJ149431.1 and KJ149356.1, respectively [26]. The germline heavy chain sequence was inferred using IMGT [50] and IgBLAST [51]. For both somatic and germline 310-18G10, the somatic 310-18G10 light chain sequence was used. Gibson cloning was then performed using the NEBuilder HiFi DNA Assembly Master Mix (NEB, cat no. E2621), using the manufacturer’s instructions following mismatch PCR to modify the bases desired. The plasmids were co-transfected into Expi293F cells at a 2:1 (HC:LC) mass ratio using ExpiFectamine 293 Reagent (Thermo Fisher Scientific, cat no. A14525). At 5 days post-transfection, the supernatant was collected, filtered using 100.0 µm Millipore Steriflip Vacuum Tube Top Filters (Millipore Sigma, cat no. SCNY00100), and the Fab was purified using a CaptureSelect CH1-XL Affinity Matrix (Thermo Fisher Scientific, cat no. 194371201L).

### Biolayer interferometry binding assay

Binding assays were performed by BLI using an Octet Red96e instrument (Sartorius) at room temperature. Briefly, biotinylated H1 stem, H3 HA, or BHA proteins at 20 µg mL^−1^ in 1× kinetics buffer (PBS at pH 7.4, 0.01% w/v BSA, and 0.002% v/v Tween 20) were loaded onto streptavidin (SA) biosensors and incubated with the indicated concentrations of Fabs. The assay consisted of five steps, each run for at least 60 seconds: (1) baseline: 1 × kinetics buffer; (2) loading: HA proteins; (3) baseline: 1× kinetics buffer; (4) association: Fab samples; and (5) dissociation: 1× kinetics buffer. Two to three Fab concentrations at 3-fold serial dilutions were used for each assay. For estimating the *K*_*D*_, a 1:1 binding model was used. In cases where 1:1 binding model did not fit well due to the contribution of non-specific binding to the response curve, a 2:1 heterogeneous ligand model was used to improve the fitting.

### Differential scanning fluorimetry assay

Thermal shift assays were performed using differential scanning fluorimetry (DSF) on the CFX Opus 96 Real-Time PCR System (Bio-Rad, cat no. 12011319). SYPRO Orange Protein Gel Stain (5,000× concentrate in dimethyl sulfoxide) (Thermo Fisher Scientific, cat no. S6650). Five micrograms of each Fab was used per assay in a total volume of 25 µL with SYPRO Orange Protein Gel Stain at 1× in PBS in a Hard-Shell 96-Well PCR Plate (Bio-Rad, cat no. HSP9601). The assay was performed from 10 to 95 °C in increments of 0.5 °C for 10 s plus plate reading using the Förster Resonance Energy Transfer channel of the instrument. Output files were analyzed with R.

### Microneutralization assay

MDCK-SIAT1 were seeded overnight in a 96-well plate. After reaching 100% confluency, MDCK-SIAT1 cells were washed once with 1× PBS. Minimal essential media (MEM, Thermo Fisher Scientific, cat. no 12492013) containing 25 mM 4-(2-hydroxyethyl)−1-piperazineethanesulfonic acid (HEPES, Thermo Fisher Scientific, cat. no 15630080) was then added to the cells. IgGs were serially diluted 2-fold starting from 100 μg mL^−1^ and mixed with 100 TCID_50_ (median tissue culture infectious dose) of viruses at equal volume and incubated at 37 °C for 1 h. Subsequently, the mixture was inoculated into cells and incubated at 37°C for another hour. Supernatants were replaced with MEM containing 25 mM HEPES and 1 g mL^−1^ TPCK-trypsin (Thermo Fisher Scientific, cat. no 20233). After incubation, Antibodies were added back to the supernatants at the same concentrations as before the medium replacement. Each antibody condition was performed in duplicate within the same plate. Plates were incubated at 37 °C for 48 h, and virus presence was assessed by cytopathic effect to determine the minimum inhibitory concentration.

### Sequence alignment

The heavy chain sequences of CR9114, 1000-3B05, and 1009-3E06 were aligned to the IGHV1-69 germline sequence by MAFFT (v7.526) [60].

## Quantification and statistical analysis

Standard deviation for *K*_*D*_ estimation, as well as *K*_*D*_ itself, was computed by Octet analysis software 9.0. All statistical tests and correlation coefficients were computed in R.

## Supporting information

S1 Fig Occurrence frequency of each mutation before sorting. Heatmaps showing the pre-sorting frequency of each mutation in each deep mutational scanning experiment. “H1”, “H3”, and “BHA” indicate H1 stem, H3/Sing16 HA, and B/Lee40 HA, respectively. The data underlying this Figure can be found in S1 Data.
(TIFF)

S2 Fig Correlations between replicates and expression score comparisons during sort. **(A)** Correlation of log_10_ *K*_*D,app*_ values between replicates. **(B)** Correlation of −Δlog_10_ *K*_*D,app*_ values between replicates. **(C)** Correlation of expression scores with −Δlog_10_ *K*_*D,app*_ values. Each teal, blue, or orange dot placed at the coordinate values represents a missense, nonsense, or silent mutation, respectively. “H1”, “H3”, and “BHA” indicate H1 stem, H3/Sing16 HA, and B/Lee40 HA, respectively. The data underlying this Figure can be found in S1 Data.
(TIFF)

S3 Fig Correlation of Tite-Seq data in this study with that of a published study and with biolayer interferometry (BLI) data. **(A)** The correlation of −Δlog_10_ *K*_*D,app*_ values between this study and a previously study by Phillips and colleagues [16]. Each data point represents a mutation for which data is available in both datasets. The Pearson correlation coefficient (*R*) is indicated. **(B)** The log_10_ *K*_*D*_ values determined by biolayer interferometry (BLI) are plotted against the log_10_ *K*_*D,app*_ values determined by Tite-Seq in the deep mutational scanning experiments. Each data point represents a mutation. Mutations with no detectable binding in BLI are assigned a log_10_ *K*_*D*_ value of −5. The genetic background of each mutation (i.e., germline or somatic CR9114) and the binding target (i.e., H1 stem, H3/Sing16 HA, or B/Lee40 HA) are color-coded. Pearson correlation coefficient (*R*) is indicated. The data underlying this Figure can be found in S3 Data.
(TIFF)

S4 Fig Binding kinetics of germline and somatic CR9114 mutants against H1 stem. The binding kinetics of the indicated **(A)** germline and **(B)** somatic CR9114 mutants in Fab format against H1 stem were measured by biolayer interferometry. The Y-axis indicates the signal response. Blue lines represent the response curve, and red lines represent the best fit model (1:1 binding model or 2:1 heterogeneous ligand model, see Methods). Binding kinetics were measured for two to three concentrations of Fab at 3-fold dilution. The dissociation constants (*K*_D_) and the goodness-of-fit values (*R*^2^) are shown. The data underlying this Figure can be found in S4 Data.
(TIFF)

S5 Fig Binding kinetics of somatic CR9114 mutants against H3 HA and BHA. The binding kinetics of the indicated somatic CR9114 mutants in Fab format against **(A)** H3 HA and **(B)** BHA were measured by biolayer interferometry. The Y-axis indicates the signal response. Blue lines represent the response curve, and red lines represent the best-fit model (1:1 binding model or 2:1 heterogeneous ligand model, see Methods). Binding kinetics were measured for two to three concentrations of Fab at 3-fold dilution. The dissociation constants (*K*_*D*_) and the goodness-of-fit values (*R*^2^) are shown. The data underlying this Figure can be found in S4 Data.
(TIFF)

S6 Fig Distribution of missense mutations effects. The distribution of −Δlog_10_ *K*_*D,app*_ values for each deep mutational scanning experiment is shown as a violin plot. “H1”, “H3”, and “BHA” indicate H1 stem, H3/Sing16 HA, and B/Lee40 HA, respectively. The data underlying this Figure can be found in S1 Data.
(TIFF)

S7 Fig Measuring the thermal stability of germline and somatic CR9114 through a thermal shift assay. The first differential curves for the relative fluorescence unit (RFU) with respect to temperature are shown for germline CR9114 (green) and somatic CR9114 (purple). The reported *T*_m_ is an average of three independent biological replicates. The data underlying this Figure can be found in S5 Data.
(TIFF)

S8 Fig Correlation of mutational effects on binding affinity across different deep mutational scanning experiments for amino acid mutations that are one nucleotide different from the wild type. **(A–F)** The −Δlog_10_ *K*_*D,app*_ value of each mutation was compared between the deep mutational scanning experiments of **(A)** the germline CR9114 against H1 stem and the somatic CR9114 against H1 stem, **(B)** the germline CR9114 against H1 stem and the somatic CR9114 against H3 HA, **(C)** the germline CR9114 against H1 stem and the somatic CR9114 against BHA, **(D)** the somatic CR9114 against H1 stem and the somatic CR9114 against H3 HA, **(E)** the somatic CR9114 against H1 stem and the somatic CR9114 against BHA, **(F)** the somatic CR9114 against H3 HA and the somatic CR9114 against BHA. Each data point represents a mutation. Pearson correlation coefficients for the mutations in the CDRs and the DE loop (blue) as well as for those in the framework regions (FWRs, red) are indicated. Only those amino acid mutations that are one-nucleotide different from the wild type (i.e., germline or somatic CR9114) are included here. “H1”, “H3”, and “BHA” indicate H1 stem, H3/Sing16 HA, and B/Lee40 HA, respectively. The data underlying this Figure can be found in S1 Data.
(TIFF)

S9 Fig Correlation between the frequency of somatic hypermutations observed among known IGHV1-69 HA stem antibodies and their effects on germline CR9114 binding to H1 stem. The occurrence frequency of each somatic hypermutation among the 160 known IGHV1-69 HA stem antibodies [24] is plotted against their corresponding −Δlog_10_ *K*_*D,app*_ value from the deep mutational scanning experiment of CR9114 against H1 stem. Points in red represent somatic mutations that are present in CR9114. The data underlying this Figure can be found in S3 Data.
(TIFF)

S10 Fig Binding kinetics of IGHV1-69 antibody somatic and germline 310-18G10 mutants against H1 stem. The binding kinetics of the indicated somatic and germline mutants of 310-18G10 in Fab format against H1 stem were measured by biolayer interferometry. The Y-axis indicates the signal response. Blue lines represent the response curve, and red lines represent the best fit model (1:1 binding model or 2:1 heterogeneous ligand model, see Methods). Binding kinetics were measured for three concentrations of Fab at 3-fold dilution. The dissociation constants (*K*_*D*_) and the goodness-of-fit values (*R*^2^) are shown. The data underlying this Figure can be found in S4 Data.
(TIFF)

S11 Fig Sequence alignments of selected IGHV1-69 HA stem antibodies. Somatic hypermutations are highlighted in red. Residues of interest are labeled.
(TIFF)

S12 Fig Impacts of incubation time and probe depletion on median fluorescence intensity (MFI). **(A)** MFI is plotted for each sample according to concentration of HA probe and mutant library identity (germline or somatic CR9114). The color of each point denotes the incubation time: 1 h, 2 h, or 24 h. **(B)** The MFI is shown for pre-depleted (pink) and depleted (black) conditions for 1 h incubations. MFI is normalized to each respective unstained mutant library background signal for each time point. The data underlying this Figure can be found in S6 Data.
(TIFF)

S1 Table Forward primers for mutant library construction. (XLSX)

S2 Table Reverse primers for mutant library construction. (XLSX)

S1 Data Deep mutational scanning data. (XLSX)

S2 Data Biolayer interferometry (BLI) and neutralization data. (XLSX)

S3 Data Somatic hypermutations among IGHV1–69 HA stem antibodies and their effects on the binding affinity of germline CR9114 to H1 stem. (XLSX)

S4 Data Biolayer interferometry (BLI) data. (XLSX)

S5 Data Data from thermal shift assay. (XLSX)

S6 Data Median fluorescence intensity (MFI) values from flow cytometry analysis. (XLSX)

## Abbreviations

BLI: biolayer interferometry
BSA: bovine serum albumin
CDRs: complementarity-determining regions
DSF: differential scanning fluorimetry
Fab: fragment antigen-binding
FACS: Fluorescence-activated cell sorting
IgG: immunoglobulin G
MFI: median fluorescence intensity
MIC: minimal inhibitory concentration
MOI: multiplicity of infection
PBS: phosphate-buffered saline
PCR: polymerase chain reaction
PEAR: Paired-End reAd mergeR
RFU: relative fluorescence unit
scFv: single-chain fragment variable
