# Supplementary material for: Epistasis and pleiotropy constrain the evolution of cross-reactive breadth in a broadly neutralizing influenza antibody
Source: PLoS Biol. 2026 Sep 28;24(9):e3004021. doi: 10.1371/journal.pbio.3004021 (PMC13638050; doi:10.1371/journal.pbio.3004021)
Supplement: Supplementary file 1 [file pbio.3004021.s023.pdf]

RESPONSE TO REVIEWERS' COMMENTS

Reviewer #1 (Miriam Hood and Angela Phillips):

Dailey et al conduct a rigorous analysis of the mutational tolerance of the influenza HA stem broadly neutralizing antibody (bnAb), CR9114, and its germline precursor, providing key insight into the molecular features that constrain bnAb maturation. Using deep mutational scans and Tite-Seq, the authors conclude that due to pleiotropy and epistasis, there is a genetic barrier to IGHV1-69 HA stem bnAbs evolving cross-reactivity towards divergent HA antigens following affinity maturation against H1 HA. These findings are well supported by the data and have important implications for the development of broadly protective influenza antibody therapeutics and vaccines.

Influenza continues to place a significant burden on healthcare systems, and the risk of a novel zoonotic pandemic remains a significant concern. Understanding the molecular constraints on the development of broadly protective influenza antibodies, and therefore vaccines, is an important and currently unmet goal in the field. The authors conduct four Tite-Seq experiments, measuring the effects of all possible single amino acid mutations in the heavy chain variable region of the germline CR9114 on affinity for the H1 stem, and of the somatic CR9114 on affinity for the H1 stem, H3, and FluB. They present several key findings - 1) mutational tolerance is higher in somatic CR9114 compared to the germline, 2) mutational tolerance in somatic CR9114 is highest for H1 stem affinity and lowest for HA B affinity, 3) the impact of mutations on affinity followed a hierarchical scheme that correlates with binding affinity (i.e., mutations in CR9114 that are beneficial or neutral for H1 affinity are deleterious for H3 and FluB affinity), and 4) many IGHV1-69 anti-stem antibodies have at least one somatic mutation that reduces binding affinity in the CR9114 sequence background. These findings are well-supported by the data; however, the authors' claim that this provides a mechanistic understanding of the genetic barriers to developing broadly protective vaccines could be better supported by additional analyses and discussion as noted below. Altogether, this work has important implications for understanding the molecular constraints on the maturation of broadly neutralizing antibodies and the development of broadly protective influenza vaccines.

Response: Thanks a lot for the positive comments.

Major comments:

1. The authors should comment on whether they expect to see differences between the stem-only and full-length HA constructs. They cite another study that measured a subset of the mutants and used full-length H1 (Phillips and Lawrence 2021) - they could look at the correlation between these data and their stem H1 data.

Response: In the revised manuscript, we have compared our Tite-Seq data with that of Phillips and Lawrence (2021). The results are shown in Figure S3A and described in the Results section:

> Lines 103-117: "A previous study by Phillips et al. [16] has performed similar Tite-Seq experiments using a mutant library of CR9114 containing all possible combinations of 16 somatic hypermutations against HAs from H1N1 A/New Caledonia/20/1999, H3N2 A/Wisconsin/67/2005, and B/Malaysia/2506/2004 HA. Because the combinatorial mutant library from Phillips et al. [16] contained single amino acid mutants of the germline and somatic CR9114, we were able to compare a small subset of their data to ours. A Pearson correlation coefficient of 0.66 was observed between their $-\Delta\log_{10} K_{D,app}$ values and ours

(Figure S3A). This correlation coefficient was lower than that between our Tite-Seq experimental replicates, likely due to the different HAs being used for H1 subtype, H3 subtype and influenza B virus. The major difference is that H1 stem was used in our present study, whereas the entire H1 HA ectodomain was used by Phillips et al. [16] Consistently, when we compared only their $-\Delta\log_{10} K_{D,app}$ values against H1 HA and ours against H1 stem, the Pearson correlation coefficient decreased to 0.32. Additionally, CR9114 in fragment antigen-binding (Fab) format was used in our present study, whereas single chain fragment variable (scFv) format was used by Phillips et al. [16] This difference may also affect the impact of a given mutation on binding affinity."

2. The authors claim that binding affinity plays a bigger role than thermostability in determining mutational tolerance (lines 115-116) based solely on the Tm of the purified Fabs. However, the purified Fab has a different structural topology than the displayed scFv, which differs from that of an endogenous BCR, and these differences in topology are known to impact antibody stability. Further, even if the germline and somatic sequences have the same stability, their stabilities do not necessarily exhibit the same mutational tolerance (ie the ddG could be quite different). Thus, thermostability may play an important role in the mutational tolerance of endogenous BCRs, which these assays cannot detect. Furthermore, they later suggest that the conformational stability of CDR H1 is required for somatic CR9114 to bind BHA (line 167), which highlights the interconnected roles of stability and binding, but they do not provide evidence of conformational stability. They should provide this evidence or soften their language.

Response: Please note that CR9114 in the Fab format was used in both our yeast display (Tite-Seq) experiments and thermostability measurements. However, we agree with this reviewer's other concerns and have revised our claim in the manuscript accordingly:

> Lines 132-136 (previously lines 115-116): "This observation showed that the difference in mutational tolerance between germline and somatic CR9114 was not due to their thermostability. While it is possible that this difference was due to the stronger binding affinity of somatic CR9114, it is also possible that the mutational stability effect, which we did not measure, was generally less deleterious in somatic CR9114."

We have also toned down the language about the conformational stability of CDR H1 in the revised manuscript:

> Lines 189-190: "this observation suggested that the conformational stability of CDR H1 may be required for somatic CR9114 to bind to BHA, but not to H1 HA and H3 HA."

3. It is unclear why the steric clash involving I73W (line 183) affects affinity for H3 and B but not for H1. Would be helpful if the authors could clarify.

Response: Unfortunately, there is no available structure of CR9114 in complex with H1 HA. Additionally, the crystal structure of CR9114 in complex with H1 stem (PDB 5CJQ, resolution = 3.6 Å) did not reach atomic resolution. As a result, we currently do not have a mechanistic explanation for why I73W affects affinity for H3 and B but not for H1. We are actually planning a follow-up study to investigate the mechanisms behind this and similar observations.

4. Because mutations observed in other IGHV1-69 stem antibodies reduce affinity in the CR9114 genetic background, the authors claim that these mutations have epistatic effects on affinity (line 199). Importantly, they do not measure these mutational effects in other genetic backgrounds to support this claim. Though these mutations may indeed be epistatic, deleterious mutations could

be tolerated if antibody affinity exceeds a threshold required to confer a selective advantage during maturation.

Response: We have added a binding experiment to demonstrate the epistatic effect in another IGHV1-69 HA stem antibody. The result is shown in Figure S10 and described in the Results section of the revised manuscript:

> Lines 229-238: "To further examine the presence of epistasis in another IGHV1-69 HA stem antibody, we focused on the somatic hypermutation V_H S35N in 310-18G10, which was previously isolated from an H5N1 vaccinee [26]. V_H S35N is highly deleterious for both germline and somatic CR9114 (Figure 2), yet it is present in 8% of IGHV1-69 HA stem antibodies (Figure S9), including 310-18G10. The binding affinity of the inferred germline of 310-18G10 (hereinafter referred to as germline 310-18G10) to H1 stem was weakened by 15% upon the addition of V_H S35N (Figure S10). At the same time, reverting V_H N35 to V_H S35 weakened the binding affinity of somatic 310-18G10 to H1 stem by almost 4-fold. Therefore, V_H S35N contributed to the affinity maturation of 310-18G10, despite being deleterious in the germline 310-18G10. These results further substantiated the present of epistasis among the binding affinity landscapes of IGHV1-69 HA stem antibodies."

5. The authors suggest that because many of the mutational effects that improve affinity to H1 do not confer breadth to H3 of Flu B, sequential acquisition of breadth is unlikely (line 244) - however, previous work has shown that multiple mutations were required for the acquisition of breadth, whereas this study only contains single mutations. Given that mutations can co-occur during maturation (or that a second permissive mutation could occur before an initial deleterious mutation is purged - DeWitt 2025), it's plausible that there are alternative paths to pan-influenza A/B bnAbs that involve larger numbers of epistatic mutations not captured in this study.

Response: This is discussed in the Discussion section of the revised manuscript:

> Lines 284-289: "At the same time, multiple somatic hypermutations may occur simultaneously. A recent study has further shown that secondary compensatory mutations can occur before an initial deleterious somatic hypermutation is purged from the germinal centers [33]. Given that multiple somatic hypermutations are required for CR9114 to acquire cross-reactivity breadth [16], it is possible that CR9114-like bnAbs can develop through alternative evolutionary trajectories involving epistatic mutations not captured in our present study."

6. In Figure 6, some deleterious mutations are present in CR9114; it would be helpful if the authors could comment on whether these mutations are among those known to require compensatory mutations and whether those compensatory mutations are present or absent in the other antibodies. Given that the authors suggest the presence of this epistasis may constrain the evolution of breadth in other influenza antibodies (lines 206-207), it would be helpful to readers to have a more detailed explanation of the observed trends. This data could also be better supported with structural comparisons when possible.

Response: We have performed an analysis focusing on V_H F29S, which is a deleterious mutation with compensatory mutations identified by Phillips and Lawrence (2021). The result is shown in Figure 6 and Figure S11, and described in the Results section of the revised manuscript:

> Lines 240-249: "As a somatic hypermutation in CR9114, V_H F29S has previously been shown to have a negative first-order effect on CR9114 binding to H1N1 A/New Caledonia/1999 HA, but it has positive epistatic interactions with V_H I52S, V_H K73I, V_H S74F, V_H T75S, and V_H S76N [16]. Similarly, we observed that V_H F29S was highly deleterious for the binding of germline CR9114 to H1 stem (Figure 2). We found that V_H F29S was present in two other IGHV1-69 HA stem antibodies, namely 1009-3B05 and 1009-3E06 (Figure 6 and Figure S11). Notably, both 1009-3B05 and 1009-3E06 were isolated from the same influenza patient and belong to the same clonotype [24, 27]. While 1009-3E06 contained one of the known compensatory mutations, V_H S76N, 1009-3B05 did not contain any (Figure S11). This observation suggested that an unknown epistatic interaction involving V_H F29S was present in 1009-3B05."

Unfortunately, structural information for 1009-3B05 and 1009-3E06 is unavailable. Therefore, we are unable to perform structural comparisons to provide a mechanistic explanation for our observation.

7. In the methods, the authors should report whether their measurements are at equilibrium, as they conducted short incubations (1h at 4˚C) and did not mention whether they accounted for ligand depletion effects.

Response: In the revised manuscript, the Methods section has been updated accordingly:

> Lines 498-511: "While we used a 1-h incubation time for probe staining, longer incubation times were also tested as part of our pilot experiments. Briefly, each yeast display library was incubated with different concentrations of PE-conjugated probes. After incubation for either 1 h, 2 h, or 24 h, samples were measured by a NovoCyte Opteon (Agilent). We saw no obvious difference in binding trends after extending the incubation time for more than 1 h (Figure S12A). Moreover, the MFI of the 1-h probe incubation was similar to that of the 24-h incubation, indicating that binding was close to equilibrium, if not already reached, after 1 h. To measure probe depletion effects, a pre-depletion sample set was prepared by incubating yeast mutant library samples with 0.1 nM to 200 nM of PE-conjugated probe for 1 h, then pelleting the yeast by centrifuging at 4000×*g* at 4°C for 5 min. To prepare the post-depletion samples, the probe-containing supernatants were removed from the pre-depletion sample yeast pellets. This probe-containing 'depleted' supernatant was applied to an equivalent number of yeast cells for 1 h to generate the post-depletion sample set. Both pre- and post-depletion samples were analyzed by a NovoCyte Opteon (Agilent). The result showed that there was no probe depletion at all the tested concentrations (Figure S12B)."

> Lines 579-580: "For this analysis, ligand depletion effects were not considered because probe depletion was not observed in a pilot experiment (Figure S12B)."

8. Throughout the paper, the authors benchmark their measurements based on Δlog KD. Do the log KDs correlate between replicates and between Tite-Seq and BLI (for the validated mutants)? If the log KD do not correlate, it would be helpful to readers to understand why.

Response: In the revised manuscript, the correlation between $\log_{10} K_D$ values between Tite-Seq replicates is shown in Figure S2A, whereas the correlation between $\log_{10} K_D$ values between Tite-Seq and BLI is shown in Figure S3B, and is described in the Results section:

> Lines 582-595: “Of note, while the log_{10} $K_{D,app}$ values (not Δlog_{10} $K_{D,app}$ values) from Tite-Seq correlated well with the K_D values from the biolayer interferometry (BLI) experiment (Pearson correlation coefficients of 0.79), the log_{10} $K_{D,app}$ values were systematically lower than the log_{10} K_D values for germline CR9114 mutants against H1 stem (below the diagonal line in Figure S3B). Similarly, while germline CR9114 is known to have a much weaker binding affinity than somatic CR9114 to H1 stem (Figure S4) [16, 59], the $K_{D,app}$ value for germline CR9114 against H1 stem was 0.3 nM, which was similar to that for somatic CR9114 against H1 stem 0.7 nM. These observations indicated that the Tite-Seq experiment for germline CR9114 mutant library against H1 stem overestimated the binding affinity. One possible explanation for this artifact is that the PE-conjugated probe for the Tite-Seq experiment of the germline CR9114 mutant library against the H1 stem was prepared much earlier than the ones for other Tite-Seq experiments in this study, including that for the somatic CR9114 mutant library against the H1 stem. There may have been a batch effect in the preparation of the PE-conjugated probe, which introduced systematic noise into the $K_{D,app}$ value measurement.”

Minor Issues:

1. Visual key in Figure 2 for brown and black squares is backwards; the brown square should have validation next to it and the black square should have SHM next to it.

Response: Fixed.

Reviewer #2:
In their manuscript Dailey and colleagues try to shed a light on the evolution of the breadth of broadly protective influenza virus anti-HA mAb CR9114. The work is interesting, but there are several points that need the authors' attention.

Response: Thank you for the supportive and constructive comments.

Major points

1) Only binding data is presented. It would be good to corroborate this with at least some neutralization data with authentic influenza viruses.

Response: We have measured the neutralization potency of germline and somatic CR9114 mutants against influenza virus A/Brisbane/59/2007 (H1N1). The result is shown in Figure 4B, and described in the Results section of the manuscript:

> Lines 179-181: “The binding affinity of germline and somatic CR9114 mutants against H1 stem also showed strong correlation (Pearson correlation coefficient of 0.85) with their neutralization activity against H1N1 A/Brisbane/59/2007 virus (Figure 4B).”

Minor points

1) Line 16 and throughout the manuscript: It should be 'influenza virus hemagglutinin'. 'influenza' on its own means the disease, not the virus.

Response: Fixed.

2) Line 18/19, line 55/56, line 215: I am not sure this statement is correct. There have been a number of antibodies reported that do crossreact between influenza A virus HA and influenza B virus HA and seem to bind to the stem. Several of them are hidden in larger studies, but they have been reported.

Response: We acknowledge that several studies have reported antibodies cross-reacting with both influenza A and B virus HAs. However, to the best of our knowledge, these antibodies were not functionally characterized in those studies. Therefore, it remains unclear whether they possess neutralizing activity and qualify as broadly neutralizing antibodies (bnAbs). As a result, we believe our statements are correct, since they specifically refer to bnAbs rather than binding antibodies alone.

> Lines 18-19 (previously lines 18-19): "CR9114 remains the only HA stem bnAb that cross-reacts with both influenza A and B viruses."
>
> Lines 56-57 (previously lines 55-56): "none of the other known HA stem bnAbs have demonstrated the ability to cross-react with both influenza A and B virus HAs."
>
> Lines 257-258 (previously line 215): "CR9114 remains the only bnAb found to date that cross-reacts with both influenza A and B virus HAs."

3) Line 21: 'influenza B virus HA'

Response: We changed this to include "virus".

4) Line 23: Please keep consistent how you abbreviate influenza B virus HA. Several versions are used.

Response: Fixed.

5) Line 28/29: Please rephrase this sentence, it is grammatically wrong.

Response: We rephrased this sentence to:

> Lines 28-29: "Influenza viruses are classified into four types (A-D), with types A and B posing major global public health concerns."

6) Line 33: The correct name for the lineages is B/Victoria/2/87-like and B/Yamagata/16/88-like.

Response: Fixed.

7) Line 34: Please defined 'COVID-19'

Response: COVID-19 is defined in the revised manuscript.

8) Line 34/35/36: Please rephrase the sentence.

Response: We rephrased this sentence to:

> Lines 35-37: “Each year, H1 and H3 subtypes of influenza A virus, along with influenza B virus, cause seasonal influenza epidemics, leading to widespread illness, absenteeism, and loss of life [4].”

9) Line 127: Please keep consistent how you spell strain names.

Response: We have checked the strain names throughput the manuscript to make sure they are consistent.

10) Line 212: Is HIV-1 meant? Also, please define the abbreviation.

Response: HIV-1 is defined in the revised manuscript.

11) Line 244: Is HA reactivity meant?

Response: We clarified this to show HA reactivity was meant.

12) Line 249: How relevant are stalk antibodies against influenza B virus. Very few have been identified…..

Response: The relevant of HA stem antibodies against influenza B virus is described in the Discussion section of the revised manuscript:

> Lines 298-300: “Notably, although only a handful of monoclonal antibodies targeting the influenza B virus HA stem have been identified [37-39], human antibody responses to the influenza B virus HA stem are prevalent and may confer protection [40, 41].”

13) Line 290: Please defined IMGT.

Response: We added a definition for IMGT is defined in the revised manuscript.

14) Line 295: stop, not STOP

Response: Fixed.

15) Line 347: Define SD-CAA.

Response: SD-CAA is defined in the revised manuscript.

16) Line 367: Which trimerization domain was used?

Response: We used the T4-foldon trimerization domain, and added this information.

17) Line 395: Define SGR-CAA.

Response: SGR-CAA is defined in the revised manuscript.

18) Line 415: Are BSA and PBS already defined?

Response: In the revised manuscript, BSA and PBS are defined during their first appearance (lines 486 and 459, respectively).

19) Line 421: Please include the actual antibiotic concentration used.

Response: We changed “1×” to “100 U mL^{-1}”, and specified the catalog number and manufacturer used.

20) Line 455: Define PEAR.

Response: PEAR is defined in the revised manuscript.

21) Line 420: Define DMSO.

Response: The full name of DMSO is spelled out in the revised manuscript.

22) Line 523: Define FRET.

Response: The full name of FRET is spelled out in the revised manuscript.
