# Supplementary material for: Epistasis and pleiotropy constrain the evolution of cross-reactive breadth in a broadly neutralizing influenza antibody
Source: PLoS Biol. 2026 Sep 28;24(9):e3004021. doi: 10.1371/journal.pbio.3004021 (PMC13638050; doi:10.1371/journal.pbio.3004021)
Supplement: Peer review history

# Editor report: Decision Letter 0

**DOI:** 10.1371/journal.pbio.3004021.r001

**Authors:** Melissa Vazquez Hernandez

17 Dec 2025

Dear Dr Wu,

Thank you for submitting your manuscript entitled "Vulnerability in the breadth evolution of an influenza broadly neutralizing antibody" for consideration as a Research Article by PLOS Biology.

Your manuscript has now been evaluated by the PLOS Biology editorial staff, as well as by an academic editor with relevant expertise, and I am writing to let you know that we would like to send your submission out for external peer review.

However, before we can send your manuscript to reviewers, we need you to complete your submission by providing the metadata that is required for full assessment. To this end, please login to Editorial Manager where you will find the paper in the 'Submissions Needing Revisions' folder on your homepage. Please click 'Revise Submission' from the Action Links and complete all additional questions in the submission questionnaire.

Once your full submission is complete, your paper will undergo a series of checks in preparation for peer review. After your manuscript has passed the checks it will be sent out for review. To provide the metadata for your submission, please Login to Editorial Manager (https://www.editorialmanager.com/pbiology) within two working days, i.e. by Dec 19 2025 11:59PM.

*IMPORTANT*: PLOS Biology will be closed for the holiday season from December 22 to January 3. During this time, we will pause inviting reviewers and will resume once we return. Thank you very much for your understanding, and we wish you a wonderful holiday season.

If your manuscript has been previously peer-reviewed at another journal, PLOS Biology is willing to work with those reviews in order to avoid re-starting the process. Submission of the previous reviews is entirely optional and our ability to use them effectively will depend on the willingness of the previous journal to confirm the content of the reports and share the reviewer identities. Please note that we reserve the right to invite additional reviewers if we consider that additional/independent reviewers are needed, although we aim to avoid this as far as possible. In our experience, working with previous reviews does save time.

If you would like us to consider previous reviewer reports, please edit your cover letter to let us know and include the name of the journal where the work was previously considered and the manuscript ID it was given. In addition, please upload a response to the reviews as a 'Prior Peer Review' file type, which should include the reports in full and a point-by-point reply detailing how you have or plan to address the reviewers' concerns.

During the process of completing your manuscript submission, you will be invited to opt-in to posting your pre-review manuscript as a bioRxiv preprint. Visit http://journals.plos.org/plosbiology/s/preprints for full details. If you consent to posting your current manuscript as a preprint, please upload a single Preprint PDF.

Feel free to email us at plosbiology@plos.org if you have any queries relating to your submission.

Kind regards,

Melissa

Melissa Vazquez Hernandez, Ph.D.

Associate Editor

PLOS Biology

mvazquezhernandez@plos.org

# Peer review: Decision Letter 1

**DOI:** 10.1371/journal.pbio.3004021.r002

**Authors:** Melissa Vazquez Hernandez

11 Feb 2026

Dear Nicholas,

Thank you for your patience while your manuscript "Vulnerability in the breadth evolution of an influenza broadly neutralizing antibody" was peer-reviewed at PLOS Biology. It has now been evaluated by the PLOS Biology editors, an Academic Editor with relevant expertise, and by two independent reviewers.

In light of the reviews, which you will find at the end of this email, we would like to invite you to revise the work to thoroughly address the reviewers' reports. As you will see below, both reviewers find the study technically rigorous and timely. However, R1 raises several concerns about interpretation, including the strength of the mechanistic claims about genetic barriers to breadth, the relative roles of affinity versus stability, the inference of epistasis from single-mutation data, and the extent to which conclusions can be generalized from CR9114 to broader vaccine design. These points would require additional analyses, clarification, and a more cautious framing of some conclusions. R2 also finds the topic interesting but mentions that the manuscript relies exclusively on binding data, and feels that at least some neutralization data with authentic influenza viruses would be important to support the biological relevance of the conclusions. In addition, R2 highlights a number of concerns about overstatements regarding A/B cross-reactive stem antibodies, as well as extensive issues with terminology, consistency, and clarity throughout the manuscript. Both mention there should be more details in the methodology. We agree with all reviewer concerns and would require some additional experimental revisions to address them, including the neutralization data suggested by R2, as we consider that this would strengthen the work.

Given the extent of revision needed, we cannot make a decision about publication until we have seen the revised manuscript and your response to the reviewers' comments. Your revised manuscript is likely to be sent for further evaluation by all or a subset of the reviewers.

In addition to these revisions, you will need to complete some formatting changes, which you will receive in a follow up email. A member of our team will be in touch with a set of requests shortly.

We expect to receive your revised manuscript within 3 months. Please email us (plosbiology@plos.org) if you have any questions or concerns, or would like to request an extension.

At this stage, your manuscript remains formally under active consideration at our journal; please notify us by email if you do not intend to submit a revision so that we may withdraw it.

**IMPORTANT - SUBMITTING YOUR REVISION**

Your revisions should address the specific points made by each reviewer. Please submit the following files along with your revised manuscript:

1. A 'Response to Reviewers' file - this should detail your responses to the editorial requests, present a point-by-point response to all of the reviewers' comments, and indicate the changes made to the manuscript.

*NOTE: In your point-by-point response to the reviewers, please provide the full context of each review. Do not selectively quote paragraphs or sentences to reply to. The entire set of reviewer comments should be present in full and each specific point should be responded to individually, point by point.

You should also cite any additional relevant literature that has been published since the original submission and mention any additional citations in your response.

2. In addition to a clean copy of the manuscript, please also upload a 'track-changes' version of your manuscript that specifies the edits made. This should be uploaded as a "Revised Article with Changes Highlighted" file type.

*Re-submission Checklist*

When you are ready to resubmit your revised manuscript, please refer to this re-submission checklist: https://plos.io/Biology_Checklist

To submit a revised version of your manuscript, please go to https://www.editorialmanager.com/pbiology/ and log in as an Author. Click the link labelled 'Submissions Needing Revision' where you will find your submission record.

Please make sure to read the following important policies and guidelines while preparing your revision:

*Published Peer Review*

Please note while forming your response, if your article is accepted, you may have the opportunity to make the peer review history publicly available. The record will include editor decision letters (with reviews) and your responses to reviewer comments. If eligible, we will contact you to opt in or out. Please see here for more details:

https://blogs.plos.org/plos/2019/05/plos-journals-now-open-for-published-peer-review/

*PLOS Data Policy*

Please note that as a condition of publication PLOS' data policy (http://journals.plos.org/plosbiology/s/data-availability) requires that you make available all data used to draw the conclusions arrived at in your manuscript. If you have not already done so, you must include any data used in your manuscript either in appropriate repositories, within the body of the manuscript, or as supporting information (N.B. this includes any numerical values that were used to generate graphs, histograms etc.). For an example see here: http://www.plosbiology.org/article/info%3Adoi%2F10.1371%2Fjournal.pbio.1001908#s5

*Blot and Gel Data Policy*

We require the original, uncropped and minimally adjusted images supporting all blot and gel results reported in an article's figures or Supporting Information files. We will require these files before a manuscript can be accepted so please prepare them now, if you have not already uploaded them. Please carefully read our guidelines for how to prepare and upload this data: https://journals.plos.org/plosbiology/s/figures#loc-blot-and-gel-reporting-requirements

*Protocols deposition*

To enhance the reproducibility of your results, we recommend that if applicable you deposit your laboratory protocols in protocols.io, where a protocol can be assigned its own identifier (DOI) such that it can be cited independently in the future. Additionally, PLOS ONE offers an option for publishing peer-reviewed Lab Protocol articles, which describe protocols hosted on protocols.io. Read more information on sharing protocols at https://plos.org/protocols?utm_medium=editorial-email&utm_source=authorletters&utm_campaign=protocols

Thank you again for your submission to our journal. We hope that our editorial process has been constructive thus far, and we welcome your feedback at any time. Please don't hesitate to contact us if you have any questions or comments.

Sincerely,

Melissa

Melissa Vazquez Hernandez, Ph.D.

Associate Editor

PLOS Biology

mvazquezhernandez@plos.org

------------------------------------

REVIEWERS' COMMENTS

------------------------------------

Reviewer #1 (Miriam Hood and Angela Phillips):

Dailey et al conduct a rigorous analysis of the mutational tolerance of the influenza HA stem broadly neutralizing antibody (bnAb), CR9114, and its germline precursor, providing key insight into the molecular features that constrain bnAb maturation. Using deep mutational scans and Tite-Seq, the authors conclude that due to pleiotropy and epistasis, there is a genetic barrier to IGHV1-69 HA stem bnAbs evolving cross-reactivity towards divergent HA antigens following affinity maturation against H1 HA. These findings are well supported by the data and have important implications for the development of broadly protective influenza antibody therapeutics and vaccines.

Influenza continues to place a significant burden on healthcare systems, and the risk of a novel zoonotic pandemic remains a significant concern. Understanding the molecular constraints on the development of broadly protective influenza antibodies, and therefore vaccines, is an important and currently unmet goal in the field. The authors conduct four Tite-Seq experiments, measuring the effects of all possible single amino acid mutations in the heavy chain variable region of the germline CR9114 on affinity for the H1 stem, and of the somatic CR9114 on affinity for the H1 stem, H3, and FluB. They present several key findings - 1) mutational tolerance is higher in somatic CR9114 compared to the germline, 2) mutational tolerance in somatic CR9114 is highest for H1 stem affinity and lowest for HA B affinity, 3) the impact of mutations on affinity followed a hierarchical scheme that correlates with binding affinity (i.e., mutations in CR9114 that are beneficial or neutral for H1 affinity are deleterious for H3 and FluB affinity), and 4) many IGHV1-69 anti-stem antibodies have at least one somatic mutation that reduces binding affinity in the CR9114 sequence background. These findings are well-supported by the data; however, the authors' claim that this provides a mechanistic understanding of the genetic barriers to developing broadly protective vaccines could be better supported by additional analyses and discussion as noted below. Altogether, this work has important implications for understanding the molecular constraints on the maturation of broadly neutralizing antibodies and the development of broadly protective influenza vaccines.

Major comments:

1.The authors should comment on whether they expect to see differences between the stem-only and full-length HA constructs. They cite another study that measured a subset of the mutants and used full-length H1 (Phillips and Lawrence 2021) - they could look at the correlation between these data and their stem H1 data.

2.The authors claim that binding affinity plays a bigger role than thermostability in determining mutational tolerance (lines 115-116) based solely on the Tm of the purified Fabs. However, the purified Fab has a different structural topology than the displayed scFv, which differs from that of an endogenous BCR, and these differences in topology are known to impact antibody stability. Further, even if the germline and somatic sequences have the same stability, their stabilities do not necessarily exhibit the same mutational tolerance (ie the ddG could be quite different). Thus, thermostability may play an important role in the mutational tolerance of endogenous BCRs, which these assays cannot detect. Furthermore, they later suggest that the conformational stability of CDR H1 is required for somatic CR9114 to bind BHA (line 167), which highlights the interconnected roles of stability and binding, but they do not provide evidence of conformational stability. They should provide this evidence or soften their language.

3.It is unclear why the steric clash involving I73W (line 183) affects affinity for H3 and B but not for H1. Would be helpful if the authors could clarify.

4.Because mutations observed in other IGHV1-69 stem antibodies reduce affinity in the CR9114 genetic background, the authors claim that these mutations have epistatic effects on affinity (line 199). Importantly, they do not measure these mutational effects in other genetic backgrounds to support this claim. Though these mutations may indeed be epistatic, deleterious mutations could be tolerated if antibody affinity exceeds a threshold required to confer a selective advantage during maturation.

5.The authors suggest that because many of the mutational effects that improve affinity to H1 do not confer breadth to H3 of Flu B, sequential acquisition of breadth is unlikely (line 244) - however, previous work has shown that multiple mutations were required for the acquisition of breadth, whereas this study only contains single mutations. Given that mutations can co-occur during maturation (or that a second permissive mutation could occur before an initial deleterious mutation is purged - DeWitt 2025), it's plausible that there are alternative paths to pan-influenza A/B bnAbs that involve larger numbers of epistatic mutations not captured in this study.

6.In Figure 6, some deleterious mutations are present in CR9114; it would be helpful if the authors could comment on whether these mutations are among those known to require compensatory mutations and whether those compensatory mutations are present or absent in the other antibodies. Given that the authors suggest the presence of this epistasis may constrain the evolution of breadth in other influenza antibodies (lines 206-207), it would be helpful to readers to have a more detailed explanation of the observed trends. This data could also be better supported with structural comparisons when possible.

7.In the methods, the authors should report whether their measurements are at equilibrium, as they conducted short incubations (1h at 4˚C) and did not mention whether they accounted for ligand depletion effects.

8.Throughout the paper, the authors benchmark their measurements based on �log KD. Do the log KDs correlate between replicates and between Tite-Seq and BLI (for the validated mutants)? If the log KD do not correlate, it would be helpful to readers to understand why.

Minor Issues:

1.Visual key in Figure 2 for brown and black squares is backwards; the brown square should have validation next to it and the black square should have SHM next to it.

------------------------------------

Reviewer #2:

In their manuscript Dailey and colleagues try to shed a light on the evolution of the breadth of broadly protective influenza virus anti-HA mAb CR9114. The work is interesting, but there are several points that need the authors' attention.

Major points

1) Only binding data is presented. It would be good to corroborate this with at least some neutralization data with authentic influenza viruses.

Minor points

1) Line 16 and throughout the manuscript: It should be 'influenza virus hemagglutinin'. 'influenza' on its own means the disease, not the virus.

2) Line 18/19, line 55/56, line 215: I am not sure this statement is correct. There have been a number of antibodies reported that do crossreact between influenza A virus HA and influenza B virus HA and seem to bind to the stem. Several of them are hidden in larger studies, but they have been reported.

3) Line 21: 'influenza B virus HA'

4) Line 23: Please keep consistent how you abbreviate influenza B virus HA. Several versions are used.

5) Line 28/29: Please rephrase this sentence, it is grammatically wrong.

6) Line 33: The correct name for the lineages is B/Victoria/2/87-like and B/Yamagata/16/88-like.

7) Line 34: Please defined 'COVID-19'

8) Line 34/35/36: Please rephrase the sentence.

9) Line 127: Please keep consistent how you spell strain names.

10) Line 212: Is HIV-1 meant? Also, please define the abbreviation.

11) Line 244: Is HA reactivity meant?

12) Line 249: How relevant are stalk antibodies against influenza B virus. Very few have been identified…..

13) Line 290: Please defined IMGT.

14) Line 295: stop, not STOP

15) Line 347: Define SD-CAA.

16) Line 367: Which trimerization domain was used?

17) Line 395: Define SGR-CAA.

18) Line 415: Are BSA and PBS already defined?

19) Line 421: Please include the actual antibiotic concentration used.

20) Line 455: Define PEAR.

21) Line 420: Define DMSO.

22) Line 523: Define FRET.

# Author comment: Author response to Decision Letter 2

**DOI:** 10.1371/journal.pbio.3004021.r003

12 Aug 2026

Attachment Submitted filename: Dailey_Response_Letter_R1v5.pdf

# Editor report: Decision Letter 2

**DOI:** 10.1371/journal.pbio.3004021.r004

**Authors:** Melissa Vazquez Hernandez

8 Sep 2026

Dear Nicholas,

Thank you for your patience while we considered your revised manuscript "Vulnerability in the breadth evolution of an influenza broadly neutralizing antibody" for publication as a Research Article at PLOS Biology. This revised version of your manuscript has been evaluated by the PLOS Biology editors, and the Academic Editor.

Based on our Academic Editor's assessment of your revision, we are likely to accept this manuscript for publication, provided you satisfactorily address the following data and other policy-related requests.

1) We routinely suggest changes to titles to ensure maximum accessibility for a broad, non-specialist readership, and to ensure they reflect the contents of the paper. I Please ensure you change both the manuscript file and the online submission system, as they need to match for final acceptance:

"Epistasis and pleiotropy constrain the evolution of cross-reactive breadth in an broadly neutralizing influenza antibody"

2) Please add the Weblink of the funding agencies in the Financial Disclosure statement in the manuscript details.

3) We do not have a word limit. Please move the references in the supplements also to the main text which can provide the readers an easier access to all information.

4) You may be aware of the PLOS Data Policy, which requires that all data be made available without restriction: http://journals.plos.org/plosbiology/s/data-availability. For more information, please also see this editorial: http://dx.doi.org/10.1371/journal.pbio.1001797

Please supply the numerical values either in the a supplementary file or as a permanent DOI’d deposition for the following figures:

Figure 2A-D, 3A-F, 4ABC, 6, S1, S2ABC, S3AB, S4AB, S5AB, S6, S7, S8A-F, S9, S10, S12

NOTE: the numerical data provided should include all replicates AND the way in which the plotted mean and errors were derived (it should not present only the mean/average values).

5) Please cite the location of the data clearly in all relevant main and supplementary Figure legends, e.g. “The data underlying this Figure can be found in S1 Data” or “The data underlying this Figure can be found in https://doi.org/10.5281/zenodo.XXXXX”

6) Please ensure that you are using best practice for statistical reporting and data presentation. These are our guidelines https://journals.plos.org/plosbiology/s/best-practices-in-research-reporting#loc-statistical-reporting and a useful resource on data presentation https://journals.plos.org/plosbiology/article?id=10.1371/journal.pbio.1002128

-- If you are reporting experiments where n ≤ 5, please plot each individual data point.

7) Please ensure that your Data Statement in the submission system accurately describes where your data can be found and is in final format, as it will be published as written there

8) Per journal policy, if you have generated any custom code during the course of this investigation, please make it available without restrictions. Please ensure that the code is sufficiently well documented and reusable, and that your Data Statement in the Editorial Manager submission system accurately describes where your code can be found. More information on our Code Policy, what and how to share can be found here: https://journals.plos.org/plosbiology/s/code-availability

Please note that we cannot accept sole deposition of code in GitHub, as this could be changed after publication. However, you can archive this version of your publicly available GitHub code to Zenodo. Once you do this, it will generate a DOI number, which you will need to provide in the Data Accessibility Statement (you are welcome to also provide the GitHub access information). See the process for doing this here: https://docs.github.com/en/repositories/archiving-a-github-repository/referencing-and-citing-content

As you address these items, please take this last chance to review your reference list to ensure that it is complete and correct. If you have cited papers that have been retracted, please include the rationale for doing so in the manuscript text, or remove these references and replace them with relevant current references. Any changes to the reference list should be mentioned in the cover letter that accompanies your revised manuscript.

In addition to these revisions, you may need to complete some formatting changes, which you will receive in a follow up email. A member of our team will be in touch with a set of requests shortly. If you do not receive a separate email within a few days, please assume that checks have been completed, and no additional changes are required.

We expect to receive your revised manuscript within two weeks.

To submit your revision, please go to https://www.editorialmanager.com/pbiology/ and log in as an Author. Click the link labelled 'Submissions Needing Revision' to find your submission record. Your revised submission must include the following:

- a cover letter that should detail your responses to any editorial requests, if applicable, and whether changes have been made to the reference list

- a Response to Reviewers file that provides a detailed response to the reviewers' comments (if applicable, if not applicable please do not delete your existing 'Response to Reviewers' file.)

- a track-changes file indicating any changes that you have made to the manuscript.

NOTE: If Supporting Information files are included with your article, note that these are not copyedited and will be published as they are submitted. Please ensure that these files are legible and of high quality (at least 300 dpi) in an easily accessible file format. For this reason, please be aware that any references listed in an SI file will not be indexed. For more information, see our Supporting Information guidelines:

https://journals.plos.org/plosbiology/s/supporting-information

*Published Peer Review History*

Please note that you may have the opportunity to make the peer review history publicly available. The record will include editor decision letters (with reviews) and your responses to reviewer comments. If eligible, we will contact you to opt in or out. Please see here for more details:

https://plos.org/published-peer-review-history/

*Press*

Should you, your institution's press office or the journal office choose to press release your paper, please ensure you have opted out of Early Article Posting on the submission form. We ask that you notify us as soon as possible if you or your institution is planning to press release the article.

*Protocols deposition*

To enhance the reproducibility of your results, we recommend that if applicable you deposit your laboratory protocols in protocols.io, where a protocol can be assigned its own identifier (DOI) such that it can be cited independently in the future. Additionally, PLOS ONE offers an option for publishing peer-reviewed Lab Protocol articles, which describe protocols hosted on protocols.io. Read more information on sharing protocols at https://plos.org/protocols?utm_medium=editorial-email&utm_source=authorletters&utm_campaign=protocols

Please do not hesitate to contact me should you have any questions.

Sincerely,

Melissa

Melissa Vazquez Hernandez, Ph.D.

Senior Editor

mvazquezhernandez@plos.org

PLOS Biology

# Editor report: Decision Letter 3

**DOI:** 10.1371/journal.pbio.3004021.r005

**Authors:** Melissa Vazquez Hernandez

14 Sep 2026

Dear Nicholas,

Thank you for the submission of your revised Research Article "Epistasis and pleiotropy constrain the evolution of cross-reactive breadth in a broadly neutralizing influenza antibody" for publication in PLOS Biology. On behalf of my colleagues and the Academic Editor, Harmit S. Malik, I am pleased to say that we can in principle accept your manuscript for publication, provided you address any remaining formatting and reporting issues. These will be detailed in an email you should receive within 2-3 business days from our colleagues in the journal operations team; no action is required from you until then. Please note that we will not be able to formally accept your manuscript and schedule it for publication until you have completed any requested changes.

Please take a minute to log into Editorial Manager at http://www.editorialmanager.com/pbiology/, click the "Update My Information" link at the top of the page, and update your user information to ensure an efficient production process.

PRESS

We frequently collaborate with press offices. If your institution or institutions have a press office, please notify them about your upcoming paper at this point, to enable them to help maximise its impact. If the press office is planning to promote your findings, we would be grateful if they could coordinate with biologypress@plos.org. If you have previously opted in to the early version process, we ask that you notify us immediately of any press plans so that we may opt out on your behalf.

We also ask that you take this opportunity to read our Embargo Policy regarding the discussion, promotion and media coverage of work that is yet to be published by PLOS. As your manuscript is not yet published, it is bound by the conditions of our Embargo Policy. Please be aware that this policy is in place both to ensure that any press coverage of your article is fully substantiated and to provide a direct link between such coverage and the published work. For full details of our Embargo Policy, please visit http://www.plos.org/about/media-inquiries/embargo-policy/.

Thank you again for choosing PLOS Biology for publication and supporting Open Access publishing. We look forward to publishing your study.

Sincerely,

Melissa

Melissa Vazquez Hernandez, Ph.D., Ph.D.

Senior Editor

PLOS Biology

mvazquezhernandez@plos.org
